# Anti-*Trypanosoma cruzi* Potential of New Pyrazole-Imidazoline Derivatives

**DOI:** 10.3390/molecules30153082

**Published:** 2025-07-23

**Authors:** Edinaldo Castro de Oliveira, Leonardo da Silva Lara, Lorraine Martins Rocha Orlando, Sarah da Costa Lanera, Thamyris Perez de Souza, Nathalia da Silva Figueiredo, Vitoria Barbosa Paes, Ana Carolina Mazzochi, Pedro Henrique Myra Fernandes, Maurício Silva dos Santos, Mirian Claudia de Souza Pereira

**Affiliations:** 1 Laboratório de Ultraestrutura Celular, Instituto Oswaldo Cruz, Fiocruz, Av. Brasil 4365 Manguinhos, Rio de Janeiro 21040-900, RJ, Brazil; castroedinaldo.07@gmail.com (E.C.d.O.); leonardosilva.lara@hotmail.com (L.d.S.L.); lorrainemartins07@hotmail.com (L.M.R.O.); sarah@lanera.com.br (S.d.C.L.); perezthamyris@gmail.com (T.P.d.S.); nathaliadasilvafigueiredo@gmail.com (N.d.S.F.); vitpaes@gmail.com (V.B.P.); 2 Laboratório de Síntese de Sistemas Heterocíclicos (LaSSH), Instituto de Física e Química (IFQ), Universidade Federal de Itajubá, Avenida BPS, 1303, Pinheirinho, Itajubá, Minas Gerais 37500-903, MG, Brazil; d2020022260@unifei.edu.br (A.C.M.); d2020009400@unifei.edu.br (P.H.M.F.); mauriciosantos@unifei.edu.br (M.S.d.S.)

**Keywords:** *Trypanosoma cruzi*, pyrazole-imidazoline, 3D culture model, chemotherapy, Chagas disease

## Abstract

Chagas disease, caused by *Trypanosoma cruzi*, poses a significant public health challenge due to its widespread prevalence, limited therapeutic options, and adverse effects associated with available medications. In this study, we developed 13 novel pyrazole-imidazoline derivatives, inspired by a previously identified cysteine protease inhibitor, and evaluated their antiparasitic activity. Our in silico analyses predicted favorable physicochemical profiles and promising oral bioavailability for these derivatives. Upon phenotypic screening, we observed that these new derivatives exhibited low cytotoxicity (CC_50_ > 100 µM) and marked efficacy against intracellular amastigotes. Derivative **1k** showed high activity (IC_50_ = 3.3 ± 0.2 µM), selectivity (SI = 73.9), and potency (pIC_50_ = 5.4). In a 3D cardiac microtissue model, **1k** significantly reduced parasite load, matching the efficacy of benznidazole (Bz) even at lower concentrations. Both **1k** and Bz effectively prevented parasite recrudescence; however, neither resulted in parasite sterility under the experimental conditions employed. The combination of **1k**–Bz yielded an additive interaction, highlighting its potential for in vivo combination therapy. While structural changes abolished cysteine protease inhibition, incorporating a CF_3_ substituent at the *para* position and excluding the amino group enhanced antiparasitic activity. These findings reinforce the promise of the pyrazole-imidazoline scaffold and support further structural optimizations to develop innovative candidates for treating Chagas disease.

## 1. Introduction

Chagas disease (CD), caused by the protozoan parasite *Trypanosoma cruzi*, represents a significant public health concern with potentially lethal outcomes, particularly in its chronic stages. Current estimates indicate that CD affects 6 to 7 million individuals globally, resulting in around 12,000 deaths annually and placing 75 million people at risk of infection [1]. The World Health Organization (WHO) has classified CD as one of the 20 neglected tropical diseases, with endemic regions in 21 Latin American countries and occurrences in Asia, Australia, Europe, and North America [2,3]. The epidemiology of CD is intricately linked to socioeconomic determinants, such as poverty, substandard housing, and inadequate access to healthcare resources, which disproportionately impact more vulnerable populations [4]. CD is characterized by two phases: acute and chronic, each with distinct symptomatology and severity profiles influenced by the host’s immune response and the stage of the infection [5]. In the chronic phase, the disease may cause severe complications, including cardiac arrhythmias and dilated cardiomyopathy, which pose a risk of sudden death or heart failure [6]. Furthermore, chronic CD can also manifest with gastrointestinal disturbances, vascular accidents, and neurological implications [7,8]. The global burden of CD is substantial, corresponding to an estimated loss of approximately 806,170 disability-adjusted life years (DALYs) and healthcare expenses exceeding US $627.56 million annually [9].

Currently, the only pharmacological treatments for CD are Benznidazole (Bz) and Nifurtimox (Nif), both of which are nitroheterocyclic compounds developed over 50 years ago. These agents demonstrate limited efficacy in the chronic phase of the disease, necessitating treatment durations of more than 60 days [10]. Their use is often complicated by significant adverse effects, frequently leading to treatment discontinuations [11,12]. A randomized clinical trial (BENEFIT) revealed that treatment with Bz could not prevent the clinical progression of chronic Chagas cardiomyopathy [13]. Monotherapy with posaconazole [14], as well as its combination with Bz [15], the prodrug of ravuconazole (E1224) [16], and the nitroimidazole fexinidazole [17], have demonstrated inadequate therapeutic outcomes in clinical trials. The limitations in efficacy and tolerability associated with current treatments, along with the therapeutic failures of potential candidates, highlight the urgent need to discover new bioactive compounds to develop more effective and safer therapeutics for CD.

Pyrazole stands out due to its structural versatility and is regarded as a privileged scaffold in medicinal chemistry. It plays a crucial role in developing various bioactive compounds that exhibit several pharmacological activities [18]. Compounds featuring a pyrazole moiety have demonstrated significant antimicrobial [19], antifungal [20], anti-inflammatory [21], antiviral [22], and antineoplastic [23] properties. Over the past few decades, the Food and Drug Administration (FDA) has approved more than 40 pyrazole-containing therapeutics [24,25], underscoring the pivotal role of this chemical framework in drug optimization strategies.

Recent studies have highlighted the antiparasitic potential of pyrazole derivatives against various infectious agents, elucidating diverse mechanisms of action. For instance, diarylpyrazole derivatives have been identified as inhibitors of CYP121A1 in *Mycobacterium tuberculosis* [26]. Furthermore, pyrazole aza macrocyclic derivatives specifically inhibit iron superoxide dismutase (FeSOD) when evaluated against *Leishmania* spp. [27]. A hybrid compound, which integrates oxadiazole and pyrazole acrylic acid, has demonstrated efficacy against *Plasmodium falciparum* in both chloroquine-resistant (RKL9) and chloroquine-sensitive (3D7) strains. This compound also effectively inhibits falcipain 2, a key enzyme in hemoglobin digestion [28].

In this context, our research group has previously identified a 5-amino pyrazole-imidazoline hybrid compound, identified as a hit compound, exhibiting anti-*T. cruzi* activity [29]. This compound inhibits the cysteine protease activity of the parasite, showing affinity for the active site of cruzain, as determined through molecular docking studies. Building upon the chemical structure of *N*-(1*H*-benzimidazol-2-yl)-1,3-dimethyl-pyrazole-4-carboxamide (3H5), a known cruzain inhibitor [30], we had previously optimized the hit compound by replacing the imidazoline ring at the 4-position of the pyrazole core with a carboxamide group. This alteration reduced antiparasitic activity [31]. However, we observed that the removal of the amino group from the hit compound and the introduction of various substituents on the phenyl ring, including 4-Cl, 4-Br and 3-Cl,4-CH_3_, enhanced antiparasitic activity [31]. These findings suggest that groups at *p*-position on the phenyl ring can improve their biological activity. Further optimization efforts included the replacement of the imidazoline moiety with a thiazoline ring, resulting in the identification of three active pyrazole-thiazoline derivatives: 2,4-diCl, 3,4-diCl, and 4-Br, all of which selectively target intracellular amastigotes [32]. Similarly, substituting the imidazoline ring with a thiadiazole yielded two active derivatives, with the 4-NO_2_ derivative demonstrating antiprotozoal efficacy comparable to Bz in controlling parasite recrudescence in vitro [33]. In this study, our goal is to further improve the activity of the compound 5-amino pyrazole-imidazoline by strategically introducing novel substituents onto the phenyl ring. This approach aims to strengthen the antiparasitic potency of the compound and increase its binding affinity for the active site of *T. cruzi* cruzain.

## 2. Results and Discussion

### 2.1. Synthesis and Chemical Analysis

**Scheme 1:**
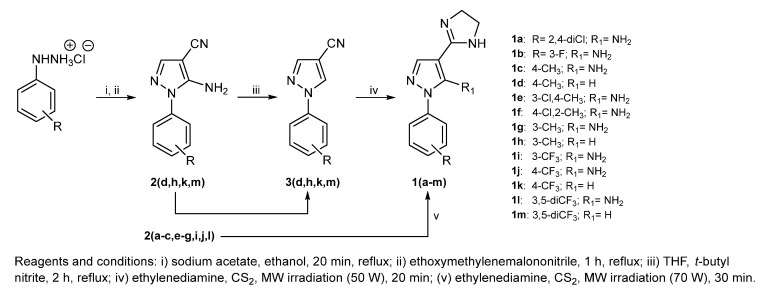
Synthetic pathway to obtain **1(a–m)**.

The desired compounds **1(a–m)** were synthesized according to the synthetic pathway outlined in Scheme 1. Firstly, arylhydrazine hydrochlorides were subjected to an acid–base reaction, using sodium acetate and ethanol as solvent, under reflux for 20 min. After that, ethoxymethylenemalononitrile was slowly added to provide a condensation reaction, followed by cyclization, while maintaining reflux for 1 h to afford **2(a–m)** in 49–99% yields. In the next step, an aprotic deamination reaction was performed to achieve four key intermediates **3(d,h,k,m)** in 87–99% yields, starting from **2(d,h,k,m)**, *t*-butyl nitrite, and tetrahydrofuran (THF), under reflux for 2 h. No characteristic bands related to the NH_2_ group were observed, confirming that the deamination reaction occurred. The syntheses of the final compounds were carried out for **2(a–c,e–g,i,j,l)** and **3(d,h,k,m)** to achieve **1(a–c,e–g,i,j,l)** and **1(d,h,k,m),** respectively, using ethylenediamine and carbon disulfide (CS_2_), under microwave irradiation conditions. In the case of **1(a–c,e–g,i,j,l)**, the power was 70 W and the reaction time was 30 min, while lower power (50 W) and shorter reaction time (20 min) were required to obtain **1(d,h,k,m).** The compounds were isolated in yields ranging from 27 to 95%. They were completely characterized by FT-IR, ^1^H NMR, ^13^C NMR, HRMS, and melting point analyses. **1(c,d,g–m)** derivatives are being published for the first time, while the synthesis of **1a** [34,35], **1b** [34,35,36], **1e** [35], and **1f** [35] have already been reported by our research group.

FT-IR analyses showed characteristic bands due to C=N stretching of the imidazoline ring at 1641–1596 cm^−1^. Bands at 3115–3064 and 2981–2847 cm^−1^ were attributed to C_sp2_-H and C_sp3_-H stretchings, respectively, while those related to C=C and C=N of aromatic rings were identified at 1583–1464 cm^−1^. ^1^H NMR and ^13^C NMR spectra confirmed that all signals corresponded to the proposed structures. ^1^H NMR spectra of **1(a−m)** revealed a singlet at 7.64–8.08 ppm that was assigned to the proton attached to C3 of the pyrazole ring. It was also identified a singlet at 8.12–8.68 ppm for deaminated derivatives **1(d,h,k,m)**, R_1_ = H, corresponding to a proton bonded to C5. Due to prototropic tautomerism in the imidazoline ring, just one signal at 3.49–3.73 ppm for two methylene (CH_2_) groups was identified. The phenyl protons were assigned according to standard coupling and integration was expected. A broad signal at 6.49–6.91 ppm assigned to protons of the amine group (NH_2_) was observed in the spectra acquired in DMSO-*d6*. On the other hand, when the analyses were carried out in methanol-*d4*, this signal did not appear. The ^13^C NMR spectra showed a signal at 49.5–50.0 ppm, attributed to two methylene (CH_2_) groups of the imidazoline ring. In case of **1a** and **1i**, the signal corresponding to CH_2_ was superposed by the solvent (methanol) at 49 ppm, as indicated by the HSQC correlation. When DMSO-*d6* was used as solvent, the signal related to CH_2_ was not identified. The carbon atoms of aromatic rings (C_sp2_) were correctly identified. In derivatives **1(c−h)** and **1(i−m)**, signals related to methyl (CH_3_) and trifluoromethyl (CF_3_), respectively, were exhibited. HRMS analyses confirmed the molecular mass of the structures. All NMR spectra are shown in the Supplementary Materials.

### 2.2. Physicochemical Properties of Pyrazole-Imidazoline Derivatives

Physicochemical properties are critical determinants in the diffusion of molecules across cellular membranes, directly impacting their biological efficacy [37]. Therefore, the physicochemical parameters were analyzed in light of Lipinski’s and Veber’s rules to predict oral bioavailability. Our findings revealed that all derivatives exhibited low molecular weights, ranging from 226.28 to 363.26 g/mol (Table 1). The lipophilicity profile, assessed using the octanol–water partition coefficient (cLogP), revealed a low-to-moderate range of hydrophobicity, with cLogP values varying from 0.21 to 1.89. The exception was derivative **1b**, which exhibited polar characteristics with a cLogP of −0.02 (Table 1). Additionally, regarding water solubility (cLogS), the derivatives demonstrated adequate hydrophilicity for systemic distribution in an aqueous environment, as evidenced by cLogS values ranging from −1.9 to −3.71 (Table 1). The derivatives exhibited a hydrogen bond donor (HBD) count of ≤2 and a hydrogen bond acceptor (HBA) count from 4 to 5 (Table 1). The topological polar surface area (tPSA) values, which predict drug diffusion across biological membranes, were recorded at 68.23 Å^2^, with exceptions noted in derivatives **1f**, **1h**, **1k**, and **1m**, which showed reduced tPSA values of 42.21 Å^2^ (Table 1). The rotatable bond (RB) count varies from 2 to 4, reflecting moderate molecular flexibility. Consequently, all derivatives comply with Lipinski’s and Veber’s rules, suggesting that the structural changes implemented may not adversely impact the drug’s oral bioavailability, thus implying a favorable pharmacokinetic profile. The physicochemical parameters of Bz, the reference drug, were also presented in Table 1.

### 2.3. Antiparasitic Effect of New Pyrazole-Imidazoline Derivatives

We evaluated thirteen new pyrazole-imidazoline derivatives for their activity against *T. cruzi*, focusing on both trypomastigotes and intracellular amastigotes. The screening uses *T. cruzi* Dm28c, genetically modified to express the luciferase enzyme (Dm28c-Luc), in conjunction with Vero cells to assess biological activity and cytotoxicity. Most of the tested pyrazole-imidazoline analogs displayed cytotoxicity values (CC_50_) exceeding 500 µM, demonstrating low toxicity. However, derivatives **1k** (CC_50_ = 243.8 ± 21.1 µM), **1l** (CC_50_ = 463.6 ± 15.2 µM), and **1m** (CC_50_ = 113.5 ± 11.9 µM) demonstrated lower CC_50_ values, but these compounds still exhibit a favorable toxicity profile (Table 2).

The new pyrazole-imidazoline derivatives showed limited activity against trypomastigotes, as evidenced by IC_50_ values exceeding 100 µM. Of the 13 derivatives analyzed, only **1e** (IC_50_ = 76.5 ± 10.3 µM) and **1k** (IC_50_ = 72.2 ± 10.3 µM) achieved values below 100 µM (Table 1), but with less activity than Bz (IC_50_ = 20.5 ± 1.8 µM). In contrast, most of the derivatives were active against intracellular amastigotes, with 84% exhibiting IC_50_ values in the range from 88.1 to 3.3 µM. The five most active derivatives were identified as follows: **1a** (IC_50_ = 40.8 ± 3.0 µM), **1d** (IC_50_ = 33.9 ± 0.8 µM), **1h** (IC_50_ = 26.1 ± 3.4 µM), **1k** (IC_50_ = 3.3 ± 0.2 µM), and **1l** (IC_50_ = 27.7 ± 2.0 µM). These compounds exhibited a selectivity index (SI) greater than 10, suggesting a favorable therapeutic window for safety. Among them, derivative **1k** stands out as a promising candidate, exhibiting biological activity comparable to Bz (IC_50_ = 2.2 ± 0.4 µM) and an SI value of 73.9. Additionally, **1k** demonstrated an IC_90_ value of 28.1 ± 0.4 µM and a potency of 5.4, which approaches the potency of Bz (pIC_50_ = 5.6). The advantageous physicochemical attributes likely contribute to its potent activity against intracellular amastigotes. Key features, including low molecular weight, optimal lipophilicity, and reduced topological polar surface area, enhance cell permeability. These factors are essential for ensuring efficient transport across the host cell membrane, enabling the compounds to reach their molecular targets within the intracellular environment of the amastigotes.

Our findings revealed that the pyrazole-imidazoline derivatives exhibited a distinct efficacy profile against extracellular and intracellular forms of *T. cruzi*. It is common to observe limited efficacy against trypomastigotes during drug screening trials for this parasite. This low responsiveness likely reflects both the mechanism of action of the compounds and the inherent characteristics of the trypomastigote form, which is marked by its non-replicative nature and low metabolic activity. Conversely, the pronounced potency demonstrated against intracellular amastigotes is particularly significant, as this life stage represents the proliferative and pathogenic stage of the parasite that supports persistent infection and contributes to chronic tissue damage in the mammalian host.

Notably, the deaminated derivative with the 4-CF_3_ substituent displayed the highest potency against intracellular amastigotes. This finding supports previous results indicating that removing the amino group and introducing electron-withdrawing groups, such as CF_3_ and halogens (Br and Cl), at the *p*-position of the phenyl ring typically enhances anti-*T. cruzi* activity. However, compounds **1a** (2,4-diCl), **1f** (4-Cl,2-CH_3_), and **1j** (4-CF_3_), exhibited low anti-*T. cruzi* activity. This unexpected outcome for these compounds may be due to the electronic and steric effects of the 5-amino group, combined with these particular aryl substituents, which could create a molecular conformation or charge distribution that is less complementary to the biological target(s). The initial optimization of the hit compound yielded three promising derivatives: 4-Cl (IC_50_ = 6 µM), 4-Br (IC_50_ = 2.75 µM), and 3-Cl,4-CH_3_ (IC_50_ = 3.58 µM) [31]. These candidates advanced to preclinical in vitro and in vivo studies, displaying favorable outcomes in more robust cellular models and in the context of preventing cardiac damage in *T. cruzi* acute infection (unpublished data). We then proceeded with **1k** for more detailed in vitro preclinical analyses.

### 2.4. Permeability and Effectiveness of **1k** in T. cruzi-Infected 3D Cardiac Microtissues

The antiparasitic efficacy of the promising **1k** derivative was evaluated using a three-dimensional cardiac microtissue model. This 3D model in our screening platform is designed to bridge the gap between in vitro and in vivo preclinical assessments, as it more accurately reflects physiological conditions compared to conventional monolayer cultures [38]. These models promote intricate cellular interactions with the extracellular matrix, which significantly influence vital biological processes, including differentiation, migration, proliferation, and cell survival. Moreover, these interactions have a significant impact on drug uptake, distribution, and cell response [39]. This is particularly relevant regarding *T. cruzi* infection, given that the heart is one of the primary targets affected in CD pathology.

**Figure 1:**
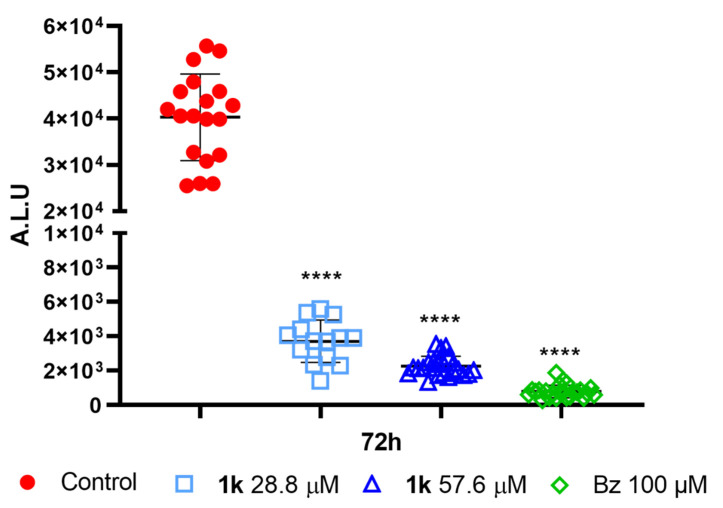
Efficacy of **1k** in three-dimensional cardiac microtissues infected by *T. cruzi*. The results are expressed in arbitrary luminescence units (A.L.U). The graph presents the mean and standard deviation of at least three independent experiments. Statistical analysis was conducted using the One-Way ANOVA method, followed by the Kruskal–Wallis test, with a significance threshold of *p* < 0.0001 (****) relative to the control group.

Therefore, the toxic effect of **1k** was assessed using 3D cardiac spheroids, which were incubated for 72 h with various concentrations of **1k**, ranging from 15.6 to 500 µM. The low toxicity profile, indicated by a CC_50_ value of 173.1 ± 6.8 µM, suggests favorable tolerability and a reduced risk of cardiotoxicity, a common reason for drug market withdrawals [40,41]. Subsequently, the antiparasitic efficacy of **1k** was investigated in *T. cruzi*-infected cardiac spheroids. Treatment with **1k** led to a significant reduction in parasite load, achieving 90% and 94% inhibition at concentrations of 28.8 µM and 57.6 µM, respectively. The **1k** treatment at 57.6 µM showed efficacy comparable to Bz at 100 µM (Figure 1), highlighting the promising potential of the **1k** derivative.

**Figure 2:**
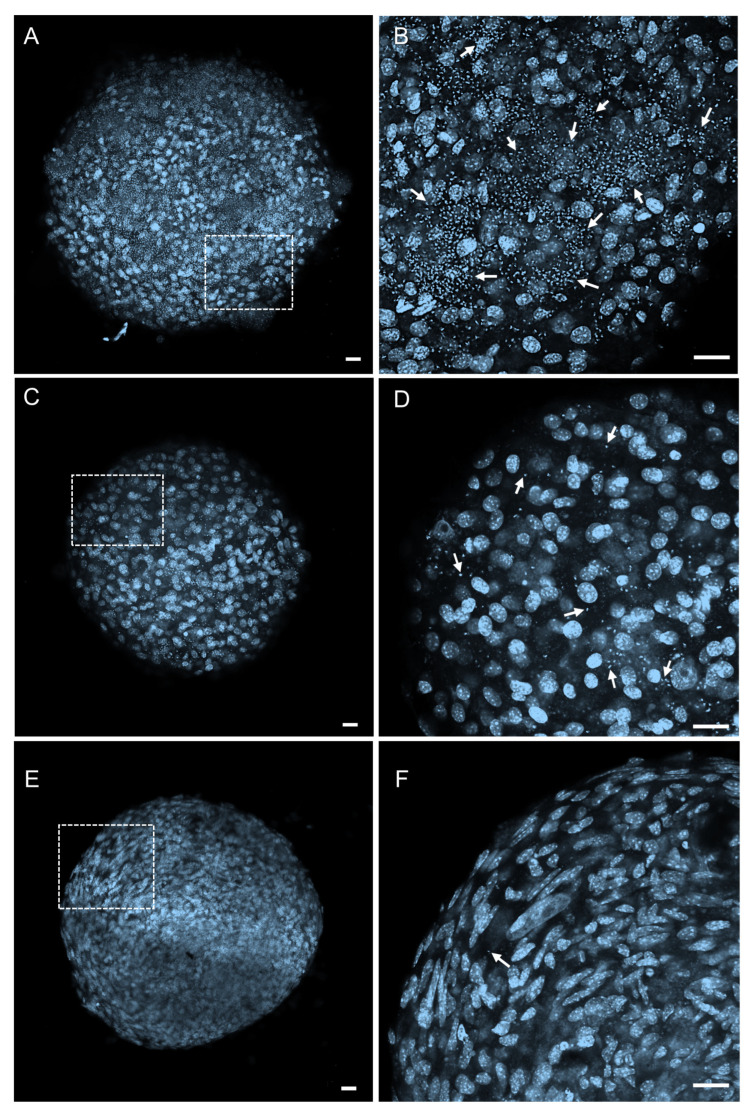
*Anti-T. cruzi* effects of **1k** and Bz in infected cardiac spheroids. (**A**,**B**) Untreated cardiac spheroid infected with *T. cruzi* for 96 h; (**C**,**D**) infected cardiac spheroid treated with **1k** at a concentration of 56.7 µM for 72 h; (**E**,**F**) treatment of infected cardiac spheroid with Bz at 100 µM for 72 h. The intracellular parasites (indicated by arrows) were visualized after DAPI staining, a DNA dye. The dashed rectangles indicate the magnified region. The spheroids were infected with Dm28c-Luc, and images were obtained using confocal microscopy (Zeiss LSM710). Scale bar = 20 µm.

The parasite load was assessed using confocal fluorescence microscopy following DAPI staining protocols. Confocal imaging revealed a substantial density of parasites in untreated 3D cardiac spheroids after 96 h of infection, with prominent amastigote nests distributed throughout the spheroid (Figure 2). Treatment with **1k** resulted in a remarkable decrease in parasite burden within spheroid cultures. At 57.6 µM concentration, **1k** exhibited a marked reduction in parasite load (Figure 2), demonstrating the compound’s effective penetration and efficacy against *T. cruzi*-infected cardiac microtissue. In spheroids treated with Bz at 100 µM, only a few parasites were observed (Figure 2).

The use of 3D culture systems in drug discovery, particularly for anticancer agents, has gained momentum [42], yet their application in antiparasitic drug screening has not been extensively explored. The translational relevance of 3D models was highlighted in HepG2 and HC-04 hepatic cell spheroids infected with *Plasmodium berghei*, where the efficacy of the antimalarial M5717 was comparable to that observed in in vivo models [43]. Furthermore, 3D models have provided valuable insights into the process of cardiac fibrosis during *T. cruzi* infection [44], demonstrating that Posaconazole treatment effectively reduces both parasite load and fibrosis by upregulating tissue inhibitor of metalloproteinases-4 (TIMP-4) [45]. Our previous investigations have also established the potential of various azole hybrids in 3D cultures, including 1,2,3-triazole [46], pyrazole-thiazoline [32], and pyrazole-thiadiazole [33]. These pyrazole derivatives successfully penetrated cardiac spheroids and significantly reduced the parasitic load. These findings emphasize the importance of 3D culture models in advancing the in vitro preclinical phase of drug development for CD.

### 2.5. Potential of **1k** in Inhibiting the Resurgence of the Parasites

**Figure 3:**
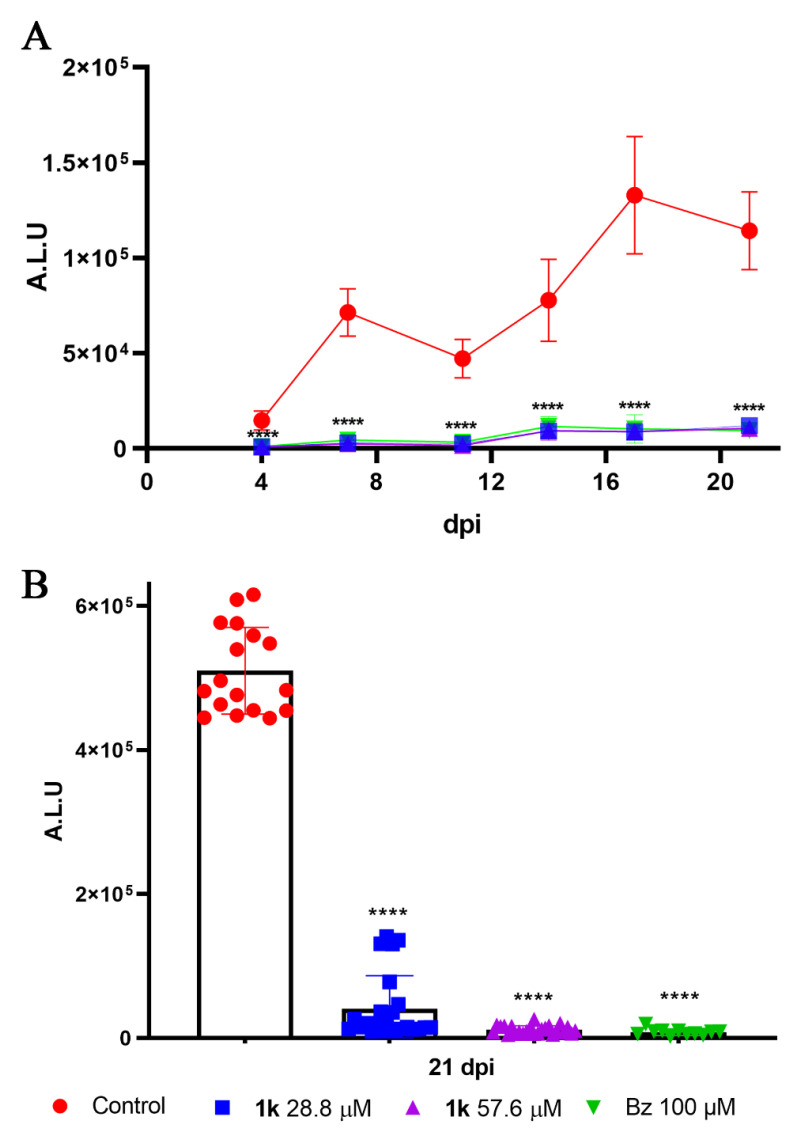
Effect of **1k** on the in vitro reversibility of infection. *T. cruzi*-infected Vero cells were treated for 10 days with **1k** and Bz, followed by an additional 10 days in culture without the drugs. (**A**) The release profile of trypomastigotes in the supernatant from untreated *T. cruzi*-infected Vero cells and those treated with **1k** (28.8 µM and 57.6 µM) and Bz (100 µM). (**B**) The infection profile of the cell monolayer at 21 days post-infection (dpi). The results are expressed in arbitrary luminescence units (A.L.U). Statistical analysis was performed using the One-Way ANOVA test, followed by the Kruskal–Wallis test, where *p* ≤ 0.0001 (****) was considered statistically significant compared to the untreated control.

Evaluating the impact of potential drug candidates on the reactivation of *T. cruzi* infection is a relevant approach to tackling drug failure. To assess the trypanocidal efficacy of **1k**, we conducted a reversibility assay, which measures the compound’s ability to inhibit the resurgence of parasitism in vitro over extended follow-up periods [47]. Thus, Vero cell cultures infected with Dm28c-Luc were exposed to **1k** for 10 days at two concentrations: 28.8 µM (IC_90_) and 57.6 µM (2-fold IC_90_). Following this treatment phase, the cultures were kept for an additional 10 days without any compound exposure. Throughout the experiment, we closely monitored the release of trypomastigotes in the culture supernatant using optical microscopy, along with assessments of parasite luminescence (A.L.U). The release of trypomastigotes into the supernatant from infected and untreated cultures (control; DMSO) began at 4 days post-infection (dpi), reaching a peak at 17 dpi (1.4 × 10^5^ A.L.U) (Figure 3A). Treatment with derivative **1k** at both 28.8 µM and 57.6 µM, as well as Bz at 100 µM, effectively suppressed parasite release until 11 dpi, with no statistically significant difference among the treatment groups. Compared to the control, the treated groups exhibited a marked reduction in trypomastigote release, maintaining an impressive 96% inhibition even after the treatment was withdrawn (Figure 3A). The infection profile of the cell monolayer was assessed at the end of the experimental assay (21 dpi). The untreated infected cultures exhibited a high parasite load (5 × 10^5^ A.L.U). Treatment with **1k** led to a significant reduction in parasite load, achieving 92% and 97.8% inhibition at concentrations of 28.8 µM (4 × 10^4^ A.L.U) and 57.6 µM (1.1 × 10^4^ A.L.U), respectively. As expected, treatment with Bz (100 µM) resulted in a decrease in the parasite load, showing a 98.4% reduction (8 × 10^3^ A.L.U) (Figure 3B).

**Figure 4:**
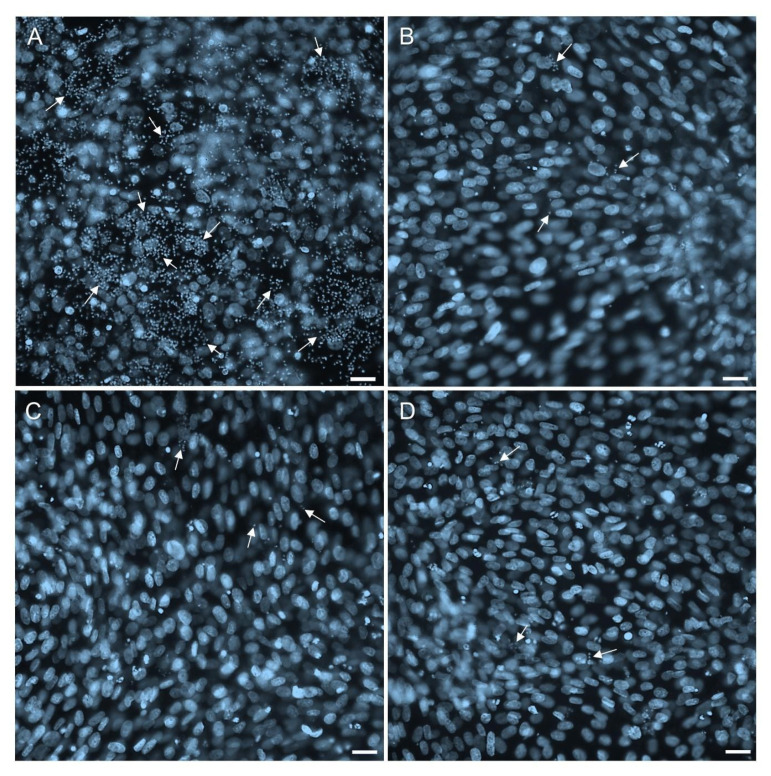
The effect of **1k** on preventing the reactivation of infection in vitro is demonstrated by fluorescence images of *T. cruzi*-infected Vero cells stained with DAPI. (**A**) Untreated cultures of Vero cells infected by *T. cruzi* (Dm28c-Luc) for 4 days. All treated groups were infected for 24 h, treated for 10 days, and then the monolayers were fixed at 21 days post-infection (dpi). (**B**,**C**) *T. cruzi*-infected Vero cell monolayers treated with **1k** at concentrations of 28.8 µM (**B**) and 57.6 µM (**C**). (**D**) Bz treatment of *T. cruzi*-infected Vero cells at 100 µM. Arrows indicate intracellular parasites. Images were acquired using the ImageXpress Micro XLS high-content screening (HCS) system (Molecular Devices, Sunnyvale, CA, USA). Bar = 20 µm.

Fluorescence microscopy analysis using a high-content imaging system (HCS) demonstrated an abundance of intracellular parasites within infected and untreated Vero cell cultures (Figure 4). DAPI staining highlighted the host cell nucleus as well as the nuclei and kinetoplasts of the parasites. Treatment with **1k** at concentrations of 28.8 µM and 57.6 µM led to a substantial reduction in parasite load, with outcomes comparable to those observed with Bz at 100 µM, even after a prolonged 10-day culture without additional treatment. By the 21 dpi mark, a few parasites were observed within the monolayers treated with **1k** or Bz (Figure 4).

Treatment with Bz and **1k** failed to achieve sterility in cultures under the tested conditions, aligning with findings from previous Bz reversibility assays [47,48]. Complete sterility was only observed at Bz concentrations 25- and 50-fold greater than the IC_50_ value after a 16-day treatment period [48]. Like Bz, pyrazole derivatives, including pyrazole-thiadiazole [33] and pyrazole-imidazoline [31], significantly reduced both intracellular amastigote and trypomastigote release in the washout assay. However, these pyrazole hybrids did not achieve sterile cure, suggesting that dormant forms may play a role in the persistence of parasitism. Washout assays are increasingly being used in the drug screening process to identify agents with undesirable trypanostatic effects early in the preclinical phases [49]. In vitro studies on infection reversibility demonstrated that posaconazole fails to inhibit parasitic recrudescence, with trypomastigotes released by day 3 post-infection, proving less effective than nitroaromatic drugs such as Bz and nifurtimox [48]. These findings are aligned with the therapeutic failure observed in the CHAGASAZOL and STOP-CHAGAS clinical trials, where posaconazole successfully suppressed parasitemia during the treatment course (60 days) but was followed by reactivation upon drug withdrawal [14,15]. Parasitic recrudescence is likely linked to the persistence and differentiation of dormant amastigote stages, as well as the trypanostatic effects of the therapeutic agents employed. This interplay may significantly influence the effectiveness of etiological treatment, making this approach paramount in the preclinical assays [48,50,51].

### 2.6. Drug Combination Effect

Combination drug therapy represents a promising avenue in the pursuit of novel therapeutic options for CD. This strategy has the potential to reduce drug dosage, shorten the overall treatment duration, mitigate adverse effects, and address issues of drug resistance by acting simultaneously on multiple targets in the pathogens [52,53].

**Figure 5:**
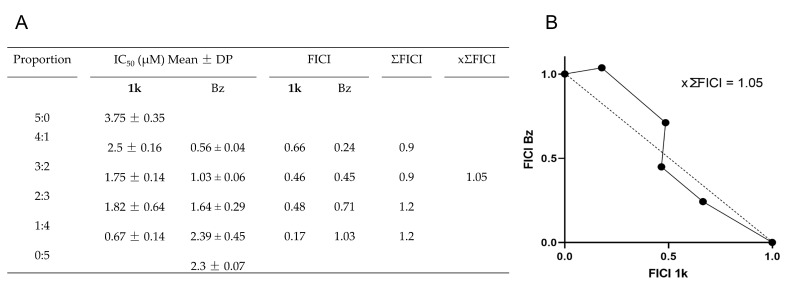
Combined effect of **1k** with Bz on intracellular amastigotes of *T. cruzi*. (**A**) The table displays the values corresponding to the fractional inhibitory concentration index (FICI), the sum of the FICI (ΣFICI), and the mean of the ΣFICI (xΣFICI). (**B**) FICI values are shown on the isobologram graph, where the dashed line indicates the range of additive values.

To enhance the antiparasitic effect of **1k**, we conducted an in vitro synergy assessment with Bz against intracellular amastigote forms of *T. cruzi*. We first established the maximum concentration for each compound and determined their respective IC_50_ values, yielding 90 µM for derivative **1k** and 61 µM for Bz. Based on these concentrations, we formulated a series of combination ratios: 5:0 (90:0 µM), 4:1 (72:12 µM), 3:2 (54:24 µM), 2:3 (36:36 µM), 1:4 (18:48.8 µM), and 0:5 (0:61 µM). The results demonstrated that the sum of fractional inhibitory concentrations (∑FICI) varied between 0.9 and 1.2, with a mean ∑FICI (x∑FICI) of 1.05, indicating an additive interaction (x∑FICI range for additivity: 0.5–4). The table in Figure 5A details the IC_50_ values, FICI, ∑FICI, and x∑FICI for each compound tested. Additionally, the isobologram presented in Figure 5B corroborates that all tested compound ratios exhibited additive interactions.

The additive interaction observed with the **1k**–Bz combination suggests that their combined effect is equivalent to the cumulative impact of each agent when administered separately [54]. This finding paves the way for further in vivo investigations into their potential in treating *T. cruzi* infections. This current assessment aims to evaluate whether combination therapy provides greater therapeutic benefits than monotherapy [53].

Previous research validates this combination strategy, demonstrating that an in vitro additive interaction of chloroquine and Bz yielded a x∑FICI of 1.4, translating to an eightfold increase in efficacy compared to Bz administered alone against a Bz-resistant Colombian strain of *T. cruzi* [55]. Likewise, the in vitro pairing of ravuconazole and amlodipine exhibited enhanced parasitic activity in a murine model, with efficacy observed even at diminished dosage of ravuconazole. This combination also significantly mitigated parasitemia reactivation after immunosuppression compared to monotherapy [56]. Further investigation on the **1k**–Bz combination in the in vivo system may contribute to the development of more potent and safer therapeutic regimens for CD.

### 2.7. Effect of **1k** on Cysteine Protease Activity

**Figure 6:**
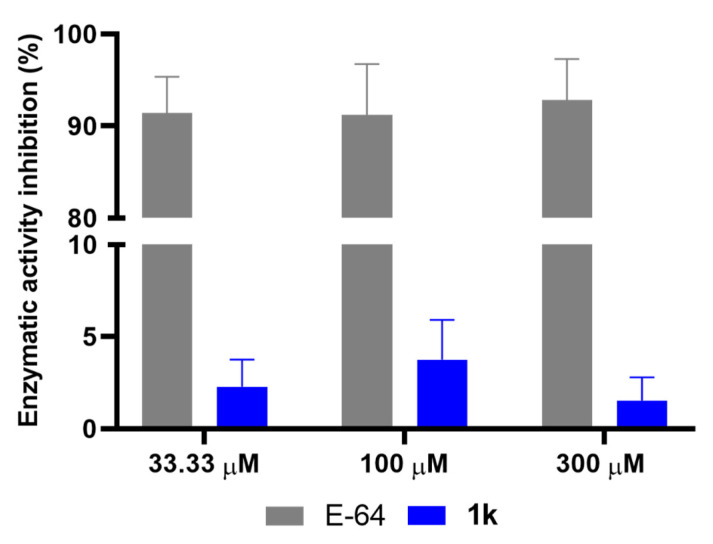
Effect of **1k** on *T. cruzi* cysteine protease activity. Protein extracts of trypomastigotes (5 µg) were incubated at 37 °C for 90 min with different concentrations of **1k** (33.33, 100, and 300 µM) and the irreversible cysteine protease inhibitor E-64 as a control. Cysteine protease activity was measured after the addition of the substrate Z-Phe-Arg-AMC (30 µM). Results are presented as a percentage of proteinase activity (%) and represent the median of three analyses. Approximately 100% of activity is relative to 56.3 × 10^5^ μmolmin^−1^ mg of protein^−1^.

In our investigation of derivative **1k** (4-CF_3_), an optimized variant of the previously identified cysteine protease inhibitor 5-amino-pyrazole-imidazoline [29], we evaluated its inhibitory potential against the cysteine protease from *T. cruzi*. Total protein extracts of trypomastigotes were incubated with concentrations of 33.3 µM, 100 µM, and 300 µM of **1k**, alongside the established cysteine protease inhibitor E-64 as a control. Following a 90 min incubation with the fluorogenic substrate Z-Phe-Arg-AMC, we assessed enzymatic activity. The results showed that **1k** exhibited poor inhibition of cysteine protease activity at only 3.76%. In contrast, the known inhibitor E-64 demonstrated a substantial inhibitory effect, reducing enzymatic activity by 92.81% (Figure 6). The incorporation of CF_3_ substitution, a functional group with a strong electron-withdrawing effect and high lipophilicity, resulted in a 5-fold enhancement in anti-*T. cruzi* activity relative to the hit compound; however, it fails to inhibit *T. cruzi* cysteine protease. The overall change in the electronic and steric profile of compound **1k**, caused by the CF_3_ group and absence of the amino group, likely led to an unfavorable interaction with the cruzain active site.

Our previous studies have demonstrated that the deamination of the hit compound, along with the substitution of the 3,5-dichloro moiety with either 4-Cl, 4-Br, or a 3-Cl/4-CH_3_ configuration, significantly enhanced antiparasitic activity while concurrently abolishing its cysteine protease inhibitory activity [31]. Furthermore, the replacement of the imidazoline ring with benzimidazole, which has been previously identified as a promising scaffold for cruzain inhibition [57], yielded no substantial improvement in enzyme inhibition, achieving only 40% inhibition [58].

Computational studies have shed light on the interactions of potent cruzain inhibitors with the enzyme’s catalytic domain [59]. The active site of cruzain is characterized by seven sub-pockets (S1—S4 and S1′—S3′) [60], with inhibitors typically interacting within the S3 to S1′ sub-pockets. The catalytic triad, comprising Cys25, His162, and Asn182, is located between the S1 and S1′ domains [61]. Furthermore, competitive inhibitors form hydrogen bond interactions with key residues, including Gly66, Asp161, and Gln19 [57]. Non-peptidic, nitrile-based compounds, which incorporate CF_3_ groups within their molecular structures, were also identified as *T. cruzi* cruzain inhibitors [62]. Additionally, compounds with a CF_3_ group at the *p*-position of the aromatic ring, such as LASSBio-1736 (a member of the hydrazide-*N*-acylhydrazone class), have demonstrated potent activity against *Leishmania* cysteine proteases [63]. The potential of CF_3_ groups in aryl chalcones has also been shown to improve interaction with the active site of cysteine proteinase B from *Leishmania (Viannia) braziliensis*, as indicated by molecular docking rankings [64]. Thus, further molecular docking studies on compound **1k** could provide deeper insights into its binding characteristics and enable structural optimization aimed at enhancing its enzyme inhibitory efficacy.

While our investigations revealed that **1k** does not significantly inhibit *T. cruzi* cysteine protease, its potent anti-amastigote activity strongly suggests an alternative mechanism of action. Given the high replicative demands of this parasitic stage, potential targets likely include enzymes involved in essential biosynthetic pathways for cell growth and division. For instance, compounds related to pyrazole scaffolds, or other nitrogen-based heterocycles, are known to target sterol biosynthesis via CYP51 [65,66]. Moreover, targeting dihydroorotate dehydrogenase (DHODH) for the inhibition of pyrimidine de novo synthesis represents a crucial strategy for managing proliferative stages, as highlighted by the efficacy of tetrahydro-1*H*,5*H*-pyrazolo [1,2-a]pyrazole-1-carboxylates against *Plasmodium falciparum* DHODH [67], an enzyme also essential for *T. cruzi* proliferation. Furthermore, interference with DNA integrity or other key metabolic processes fundamental to amastigote proliferation also represents a plausible mechanism of action. Further studies are needed to elucidate the molecular target(s) and mechanism(s) of action of **1k**, which could lead to the development of new therapeutic strategies for Chagas disease.

## 3. Materials and Methods

### 3.1. Chemistry

All commercial reagents and solvents were used as received. The melting points were determined on an Allerbest apparatus (Allerbest, Curitiba, PR, Brazil). FT–IR spectra were recorded on a PerkinElmer Spectrum 100 spectrometer equipped with an ATR diamond-ZnSe apparatus (PerkinElmer, Waltham, MA, USA), with wavenumber expressed in cm^−1^, involving 16 scans and a resolution of 4 cm^−1^. NMR spectra were recorded on a Bruker Avance (Bruker, Rheinstetten, Germany), 400 MHz or 500 MHz, at 298 K, in deuterated methanol (MeOD-*d4*) or deuterated dimethyl sulfoxide (DMSO-*d6*). Chemical shifts (δ) are expressed in parts per million (ppm) and coupling constants (*J*) in Hertz (Hz). The HRMS analyses were performed using a Q-TOF Bruker Spectrometer (Bruker, Bremen, Germany), with electrospray ionization (ESI), a capillary of 4.0 kV, an end plate offset of 4.0 kV, a source temperature of 150 °C, a dry gas administered at 4.0 l/min, and a desolvation temperature of 200 °C. The intermediates 5-amino-1-aryl-1*H*-pyrazole-4-carbonitriles **2(a−m)** and 1-aryl-1*H*-pyrazole-4-carbonitriles **3(d,h,k,m)** were synthesized following the experimental procedure previously described by our research group [68].

*5-amino-1-(2,4-dichlorophenyl)-4-(4,5-dihydro-1H-imidazol-2-yl)-1H-pyrazole (**1a**)*. Yield: 27%; color: beige; mp.: 167–169 °C; FT-IR (cm^−1^): 3385, 3259, 3115, 2934, 2853, 1608, 1581, 1565, 1532, 1484; ^1^H NMR (500 MHz, methanol-*d4*): *δ* = 7.74 (s, 1H), 7.73 (d, *J* = 2.2 Hz, 1H), 7.53 (dd, *J* = 8.5;2.2 Hz, 1H), 7.49 (d, *J* = 8.5 Hz, 1H), 3.71 (s, 4H); ^13^C NMR (124 MHz, methanol-*d4*): *δ* = 162.2, 150.4, 140.9, 137.8, 135.3, 134.9, 132.5, 131.6, 129.7, 93.3, 49.5; HRMS (ESI): *m*/*z* [M + H]^+^: calcd.: 296.0470; found: 296.0475.

*5-amino-1-(3-fluorophenyl)-4-(4,5-dihydro-1H-imidazol-2-yl)-1H-pyrazole (**1b**)*. Yield: 47%; color: yellow; mp.: 164–166 °C; FT-IR (cm^−1^): 3369, 3257, 3162, 2934, 2861, 1601, 1567, 1520, 1492; ^1^H NMR (400 MHz, DMSO-*d6*): *δ* = 7.71 (s, 1H), 7.56–7.52 (m, 1H), 7.49–7.43 (m, 2H), 7.22–7.20 (m, 1H), 6.72 (s, 2H), 3.50 (s, 4H); ^13^C NMR (100 MHz, DMSO-*d6*): *δ* = 162.1 (d, *J* = 243.0 Hz), 159.9, 147.6, 140.0 (d, *J* = 10.0 Hz), 138.7, 131.0 (d, *J* = 9.0 Hz), 118.1 (d, *J* = 3.0 Hz), 113.2 (d, *J* = 21.0 Hz), 109.4 (d, *J* = 25.0 Hz), 94.7; HRMS (ESI): *m*/*z* [M + H]^+^: calcd.: 246.1155; found: 246.1163.

*5-amino-1-(4-methylphenyl)-4-(4,5-dihydro-1H-imidazol-2-yl)-1H-pyrazole (**1c**)*. Yield: 50%; color: yellow; mp.: 214–216 °C; FT-IR (cm^−1^): 3258, 3192, 3095, 2928, 2867, 1598, 1561, 1511, 1492; ^1^H NMR (400 MHz, DMSO-*d6*): *δ* = 7.65 (s, 1H), 7.45 (d, *J* = 8.3, 2H), 7.31 (d, *J* = 8.3, 2H), 6.49 (s, 2H), 3.49 (s, 4H), 2.35 (s, 3H); ^13^C NMR (100 MHz, DMSO-*d6*): *δ* = 160.0, 147.2, 138.0, 136.1, 136.0, 129.6, 122.5, 94.2, 20.4; HRMS (ESI): *m*/*z* [M + H]^+^: calcd.: 242.1406; found: 242.1412.

*1-(4-methylphenyl)-4-(4,5-dihydro-1H-imidazol-2-yl)-1H-pyrazole (**1d**)*. Yield: 28%; color: light yellow; mp.: 232–234 °C; FT-IR (cm^−1^): 3144, 3107, 3077, 2932, 2847, 1631, 1563, 1521, 1482; ^1^H NMR (500 MHz, methanol-*d4*): *δ* = 8.50 (s, 1H), 8.02 (s, 1H), 7.62 (d, *J* = 8.3 Hz, 2H), 7.31 (d, *J* = 8.3 Hz, 2H), 3.71 (s, 4H), 2.38 (s, 3H); ^13^C NMR (125 MHz, methanol-*d4*): *δ* = 161.1 140.9, 138.7, 138.6, 131.1, 128.8, 120.5, 115.8, 49.9, 20.9; HRMS (ESI): *m*/*z* [M + H]^+^: calcd.: 227.1297; found: 227.1290.

*5-amino-1-(3-chloro-4-methylphenyl)-4-(4,5-dihydro-1H-imidazol-2-yl)-1H-pyrazole (**1e**)*. Yield: 85%; color: light yellow; mp.: 238–240 °C; FT-IR (cm^−1^): 3384, 3261, 3215, 3114, 2924, 2853, 1602, 1581, 1566, 1523, 1501, 1479; ^1^H NMR (500 MHz, DMSO-*d6*): *δ* = 7.68 (s, 1H); 7.62 (s, 1H), 7.49 (s, 1H), 7.48 (s, 1H), 6.64 (s, 2H), 3.50 (s, 4H), 2.37 (s, 3H); ^13^C NMR (125 MHz, DMSO-*d6*): *δ* = 159.9, 147.6, 138.5, 137.4, 133.7, 133.4, 131.5, 122.5, 121.0, 94.6, 19.0; HRMS (ESI): *m*/*z* [M + H]^+^: calcd.: 276.1016; found: 276.1018.

*5-amino-1-(4-chloro-2-methylphenyl)-4-(4,5-dihydro-1H-imidazol-2-yl)-1H-pyrazole (**1f**)*. Yield: 73%; color: yellow; mp.: 205–206 °C; FT-IR (cm^−1^): 3410, 3260, 3205, 3123, 2926, 2851, 1596, 1570, 1520, 1482; ^1^H NMR (500 MHz, DMSO-*d6*): *δ* = 7.64 (s, 1H), 7.51 (d, *J* = 2.3 Hz, 1H), 7.39 (dd, *J* = 8.4; 2.3 Hz, 1H), 7.28 (d, *J* = 8.4 Hz, 1H), 6.27 (s, 2H), 3.49 (s, 4H), 2.05 (s, 3H); ^13^C NMR (125 MHz, DMSO-*d6*): *δ* = 160.0, 148.3, 138.1, 138.0, 135.6, 133.1, 130.5, 129.4, 126.5, 92.9, 16.9; HRMS (ESI): *m*/*z* [M + H]^+^: calcd.: 276.1016; found: 276.1026.

*5-amino-1-(3-methylphenyl)-4-(4,5-dihydro-1H-imidazol-2-yl)-1H-pyrazole (**1g**)*. Yield: 57%; color: light yellow; mp.: 204–205 °C; FT-IR (cm^−1^): 3243, 3110, 2927, 2854, 1599, 1586, 1560, 1518, 1486, 1464; ^1^H NMR (400 MHz, methanol-*d4*): *δ* = 7.67 (s, 1H), 7.41 (t, *J* = 7.7 Hz, 1H), 7.35 (s, 1H), 7.31 (d, *J* = 7.7 Hz, 1H), 7.25 (d, *J* = 7.7 Hz, 1H), 3.64 (s, 4H), 2.41 (s, 3H); ^13^C NMR (100 MHz, methanol-*d4*): *δ* = 162.7, 148.6, 141.1, 139.8, 139.1, 130.5, 129.9, 125.9, 122.4, 95.5, 49.7, 21.4; HRMS (ESI): *m*/*z* [M + H]^+^: calcd.: 242.1406; found: 242.1414.

*1-(3-methylphenyl)-4-(4,5-dihydro-1H-imidazol-2-yl)-1H-pyrazole (**1h**)*. Yield: 95%; color: light yellow; mp.: 190–192 °C; FT-IR (cm^−1^): 3258, 3147, 3064, 2937, 2869, 1620, 1590, 1569, 1493; ^1^H NMR (400 MHz, methanol-*d4*): *δ* = 8.54 (s, 1H), 8.03 (s, 1H), 7.60 (s, 1H), 7.54 (d, *J* = 7.6 Hz, 1H), 7.38 (t, *J* = 7.6 Hz, 1H), 7.20 (d, *J* = 7.6 Hz, 1H), 3.71 (s, 4H), 2.42 (s, 3H); ^13^C NMR (100 MHz, methanol-*d4*): *δ* = 161.1, 141.1, 141.1, 140.8, 130.5, 129.2, 128.8, 121.0, 117.6, 116.0, 49.9, 21.4; HRMS (ESI): *m*/*z* [M + H]^+^: calcd.: 227.1297; found: 227.1302.

*5-amino-1-(3-trifluoromethylphenyl)-4-(4,5-dihydro-1H-imidazol-2-yl)-1H-pyrazole (**1i**)*. Yield: 38%; color: yellow; mp.: 213–215 °C; FT-IR (cm^−1^): 3392, 3258, 3205, 3117, 2928, 2860, 1597, 1573, 1523, 1490; ^1^H NMR (500 MHz, methanol-*d4*): *δ* = 7.88 (s, 1H), 7.87–7.85 (m, 1H), 7.76–7.73 (m, 3H), 3.66 (s, 4H); ^13^C NMR (125 MHz, methanol-*d4*): *δ* = 162.5, 149.2, 140.8, 140.0, 132.9 (quartet, *J* = 32.5 Hz), 131.7, 128.4, 126.2, 125.5 (quartet, *J* = 3.7 Hz), 125.1 (quartet, *J* = 270 Hz), 121.9 (quartet, *J* = 3.7 Hz), 95.8; HRMS (ESI): *m*/*z* [M + H]^+^: calcd.: 296.1123; found: 296.1113.

*5-amino-1-(4-trifluoromethylphenyl)-4-(4,5-dihydro-1H-imidazol-2-yl)-1H-pyrazole (**1j**)*. Yield: 59%; color: light yellow; mp.: 269–270 °C; FT-IR (cm^−1^): 3284, 3220, 3083, 2942, 2873, 1606, 1565, 1533, 1513, 1486; ^1^H NMR (400 MHz, DMSO-*d6*): *δ* = 7.89–7.84 (m, 4H), 7.76 (s, 1H), 6.80 (s, 2H), 3.51 (s, 4H); ^13^C NMR (100 MHz, DMSO*-d6*): *δ* = 159.8, 147.9, 141.8, 139.2, 126.4 (quartet, *J* = 32.0 Hz), 126.4 (quartet, *J* = 3.7 Hz), 124.0 (quartet, *J* = 270 Hz), 122.3, 94.9; HRMS (ESI): *m*/*z* [M + H]^+^: calcd.: 296.1123; found: 296.1129.

*1-(4-trifluoromethylphenyl)-4-(4,5-dihydro-1H-imidazol-2-yl)-1H-pyrazole (**1k**)*. Yield: 76%; color: white; mp.: 253–255 °C; FT-IR (cm^−1^): 3156, 3110, 3074, 2940, 2876, 1618, 1570, 1529, 1480; ^1^H NMR (500 MHz, methanol-*d4*): *δ* = 8.68 (s, 1H), 8.08 (s, 1H), 8.00 (d, *J* = 8.5 Hz, 2H), 7.82 (d, *J* = 8.5 Hz, 2H), 3.72 (s, 4H); ^13^C NMR (125 MHz, methanol-*d4*): *δ* = 160.8, 143.5, 142.0, 129.9 (quartet, *J* = 33.0 Hz), 128.9, 128.0 (quartet, *J* = 3.7 Hz), 125.4 (quartet, *J* = 270.0 Hz), 120.4, 116.9, 50.0; HRMS (ESI): *m*/*z* [M + H]^+^: calcd.: 281.1014; found: 281.1019.

*5-amino-1-((3,5-ditrifluoromethyl)phenyl)-4-(4,5-dihydro-1H-imidazol-2-yl)-1H-pyrazole (**1l**)*. Yield: 85%; color: yellow; mp.: 192–194 °C; FT-IR (cm^−1^): 3396, 3262, 3224, 3126, 2942, 2849, 1599, 1583, 1521, 1467; ^1^H NMR (400 MHz, DMSO-*d6*): *δ* = 8.26 (s, 2H), 8.10 (s, 1H), 7.82 (s, 1H), 6.91 (s, 2H), 3.52 (s, 4H); ^13^C NMR (100 MHz, DMSO-*d6*): *δ* = 159.7, 148.2, 140.1, 139.8, 131.2 (quartet, *J* = 33.0 Hz), 122.8 (quartet, *J* = 271 Hz), 122.3 (d, *J* = 3.0 Hz), 119.7 (quintet, *J* = 3.7 Hz), 95.5; HRMS (ESI): *m*/*z* [M + H]^+^: calcd.: 364.0991; found: 364.0985.

*1-((3,5-ditrifluoromethyl)phenyl)-4-(4,5-dihydro-1H-imidazol-2-yl)-1H-pyrazole (**1m**)*. Yield: 89%; color: white; mp.: 199–201 °C; FT-IR (cm^−1^): 3321, 3107, 3034, 2981, 2888, 1641, 1573, 1494; ^1^H NMR (500 MHz, methanol-*d4*): *δ* = 8.81 (s, 1H), 8.45 (s, 2H), 8.12 (s, 1H), 7.96 (s, 1H), 3.73 (s, 4H); ^13^C NMR (125 MHz, methanol-*d4*): *δ*= 160.6, 142.4, 142.1, 134.1 (quartet, *J* = 33.7 Hz), 129.2, 124.4 (quartet, *J* = 270 Hz), 121.2 (quintet, *J* = 3.7 Hz), 120.2 (d, *J* = 3.7 Hz), 117.3, 49.9; HRMS (ESI): *m*/*z* [M + H]^+^: calcd.: 349.0882; found: 349.0871.

### 3.2. In Silico Analysis

The physicochemical properties of the compounds were assessed using Osiris DataWarrior software version 5.5.0 [69]. All Simplified Molecular Input Entry Line System (SMILES) representation utilized in the in silico analysis was generated with MarvinSketch software (version 22.22).

### 3.3. Cell Culture

Vero cells, obtained from the Rio de Janeiro Cell Bank (BCRT code 0245) and maintained through weekly subculturing with a dissociation solution containing 0.25% trypsin-EDTA, initiated once the monolayer reached an 80–100% confluence. Following harvesting, the cells were washed and resuspended in RPMI 1640 medium enriched with 10% fetal bovine serum (FBS). Cultures were maintained at 37 °C in a 5% CO_2_-humidified incubator. These cultures were used for drug screening assays and for producing culture-derived trypomastigotes of *T. cruzi*.

Three-dimensional (3D) heart muscle cell cultures were obtained as previously described [44]. Briefly, heart muscle cells were isolated from 18-day-old Swiss Webster mouse fetuses [70]. The ventricular tissue was fragmented and enzymatically dissociated using a trypsin and collagenase type II solution. The isolated cells were seeded at a density of 2.5 × 10^4^ cells/well in 96-well U-bottom microplates previously coated with 1% agarose. The cells were cultured in Dulbecco’s modified Eagle Medium (DMEM) enriched with 10% FBS, 2.5 mM CaCl_2_, 1 mM L-glutamine, 2% chicken embryo extract, and a cocktail of antibiotics. The cultures were maintained at 37 °C in a 5% CO_2_ atmosphere. After a 7-day culture period, the resulting fully developed spheroids were used to assess drug-induced cardiotoxicity and evaluate their efficacy against *T. cruzi*. All animal experimentation protocols were approved by the Animal Care and Use Committee at Instituto Oswaldo Cruz (License L-017-2022-A2).

### 3.4. Trypanosoma cruzi

The genetically modified *Trypanosoma cruzi*, clone Dm28c (TcI), expressing luciferase (Dm28c-Luc), was kindly provided by Dr. Cristina Henriques from Oswaldo Cruz Institute—Fiocruz [71]. The parasites were maintained using Vero cell cultures in RPMI 1640 medium enriched with 10% FBS. At 4 days post-infection (dpi), the trypomastigotes released into the culture supernatant were harvested, subjected to centrifugation at 800× *g* for 20 min at 4 °C, and quantified with a Neubauer chamber. The isolated trypomastigotes were subsequently used for phenotypic screening, drug efficacy assessments employing a 3D cardiac model, washout experiments to evaluate reversibility, drug combination studies, and enzymatic assays.

### 3.5. Cytotoxicity Assay

Cytotoxicity assays were performed using Vero cells, which were seeded at a density of 1.5 × 10^4^ cells per well in white 96-well plates with clear bottoms. Following a 24 h adhesion period, the cultures were incubated at 37 °C in a 5% CO_2_ atmosphere. Concentrations of pyrazole-imidazoline compounds or Bz, ranging from 15.6 to 500 µM, were administered. After 72 h of incubation, cell viability was assessed by adding CellTiter-Glo reagent (Promega Corporation, Madison, WI, USA), which induces cell lysis and reacts with the released ATP to produce luminescence. The resulting luminescence was measured using a Glomax Multi-Detection plate reader (Promega Corporation, Madison, WI, USA). The half-maximal inhibitory concentration (CC_50_) for cell viability was then calculated using GraphPad Prism software version 8.2.1.

The cytotoxicity of the promising candidate was assessed using 3D cardiac spheroids, seeded at 2.5 × 10^4^ cells per well. The cultures were treated with a series of concentrations as previously described, and the cell viability was measured using luminescence following the addition of CellTiter-Glo reagent (Promega Corporation, Madison, WI, USA). A low concentration of dimethyl sulfoxide (DMSO < 1%) served as the negative control. The data shown represents the mean value accompanied by standard deviations, calculated from at least three independent experiments, each performed in duplicate.

### 3.6. Anti-T. cruzi In Vitro Assay

Drug susceptibility assays were performed on *T. cruzi* Dm28c-Luc, a genetically engineered parasite expressing luciferase. The drug screening used both trypomastigotes and intracellular amastigotes. Trypomastigotes (1 × 10^6^ parasites/well) were added to white 96-well plates with clear bottoms. Following a 24 h exposure to varying concentrations (ranging from 0.41 to 100 µM) of pyrazole-imidazoline compounds or Bz, D-luciferin solution (300 µg/mL) was added to assess parasite viability via luciferase activity. The luminescence was measured using a Glomax Multi-Detection plate reader (Promega Corporation, Madison, WI, USA).

To evaluate the effect of pyrazole-imidazoline derivatives against intracellular amastigotes, Vero cells (1.5 × 10^4^ cells/well) were cultured in a white 96-well plate with a clear bottom and subsequently infected with *T. cruzi* Dm28c-Luc at a 10:1 parasite-to-host cell ratio for 24 h. Post-infection, the cultures were treated with serial dilutions of pyrazole-imidazoline compounds or Bz ranging from 0.41 to 100 µM for 72 h at 37 °C. Parasite viability was measured by luminescence following the addition of luciferin (300 µg/mL). Background measurements (non-infected cultures incubated with luciferin, as well as luciferin-only controls) were performed to ensure the signal specifically originates from viable parasites. The IC_50_ and IC_90_ values, denoting the concentrations that achieve 50% and 90% reductions in viable parasites, respectively, were calculated using GraphPad Prism 8.2.1 software. Additionally, the selectivity index (SI) was calculated as the cytotoxic concentration (CC_50_) ratio in mammalian cells to the IC_50_ against *T. cruzi*, providing insight into the therapeutic window. Potency against intracellular amastigotes was evaluated using the formula 6—Log10 (IC_50_).

The efficacy of the promising candidate was assessed using cardiac 3D microtissues. Following a 7-day cultivation period, cardiac spheroids (2.5 × 10^4^ cells/well) were infected with *T. cruzi* Dm28c-Luc (20:1 parasites/host cell) for 24 h. After a washing step, the spheroids were treated with the promising candidate at concentrations of IC_90_ and 2-fold IC_90_ for 72 h at 37 °C, ensuring these concentrations remained within the CC_20_ range for the compound. Bz was used as a positive control at the concentration of 100 µM (corresponding to 10-fold IC_90_). At the end of the experiment, luminescence was measured using a Glomax Multi-Detection plate reader (Promega Corporation, Madison, WI, USA) after adding luciferin solution, with results reported in arbitrary luminescence units (A.L.U). To enhance reliability and provide qualitative, orthogonal validation of these luminescence readings, we employed confocal fluorescence microscopy using 4′,6′-diamidino-2-phenylindole (DAPI) staining. Thus, cardiac spheroids were fixed in 4% paraformaldehyde (PFA) in PBS for 20 min at 4 °C and stained with 10 µg/mL of DAPI. Imaging was performed using a Zeiss LSM710 confocal fluorescence microscope (Carl Zeiss, Baden–Württemberg, Germany). For all drug assays, low concentrations of DMSO (<1%) were used as a negative control. Each experimental condition was replicated in duplicates across at least three independent trials.

### 3.7. Reversibility Assay

Monolayers of Vero cells infected with *T. cruzi* (Dm28c-Luc) for 24 h were treated with concentrations corresponding to the IC_90_ and 2-fold IC_90_ of the promising candidate for 10 days. After treatment, the cultures were thoroughly washed, and parasite recrudescence was evaluated over an extended period of 10 days in RPMI medium supplemented with 10% fetal bovine serum (FBS). Culture supernatants were collected at intervals, and the luminescence signal indicative of trypomastigote release was assessed using a Glomax Multi-Detection plate reader (Promega Corporation, Madison, WI, USA) following the addition of luciferin every 2–3 days. At the experiment’s conclusion, the infection profile of the monolayer was also examined. Cells were fixed with PFA for 20 min at 4 °C, then stained with DAPI for microscopy analysis. Luminescence data were expressed in arbitrary luminescent units (A.L.U), and fluorescence images were acquired using the ImageXpress Micro XLS high-content screening (HCS) system (Molecular Devices, Sunnyvale, CA, USA).

### 3.8. Drug Combination

The isobologram approach assessed the interaction between Bz and the promising candidate in intracellular amastigotes of *T. cruzi* [72]. Vero cell cultures infected with *T. cruzi* (Dm28c-Luc) were treated for 72 h with various concentrations of combinations of Bz and the promising candidate, specifically at ratios of 5:0; 4:1; 3:2; 2:3; 1:4 and 0:5. The maximum concentration of each compound was established based on their IC_50_ values, ensuring both reached the fourth dilution in the serial dilution setup (1:3), culminating in a total of seven dilutions. After 72 h of treatment, parasite viability was measured by luminescence in a Glomax Multi-Detection plate reader (Promega Corporation, Madison, WI, USA) after incubation with luciferin. The IC_50_ values of each treatment combination were calculated individually, and the fractional inhibitory concentration index (FICI) was derived using the formula FICI = IC_50_ of the combination/IC_50_ of the compound alone. The overall FICI (ΣFICI) was calculated by summing the FICIs for both compounds in each combination group, while the average ΣFICI (xΣFICI) represented the overall mean across all combination groups. The xΣFICI values were then employed to classify the interaction as synergistic (xΣFICI ≤ 0.5), additive (0.5 < xΣFICI < 4), or antagonistic (xΣFICI > 4). The isobologram plot was generated using GraphPad Prism version 8.2.1, with averages of ΣFICI derived from three independent experiments.

### 3.9. Enzymatic Activity

Total proteins from *T. cruzi* Dm28c-Luc were extracted using a lysis buffer [100 mM Tris-HCl pH 8.0, 150 mM NaCl, 10% glycerol, 0.5% Triton X-100]. The protein concentration was quantified using the Bradford assay, with bovine serum albumin (BSA) used to establish the standard curve. For the assessment of cysteine protease activity, 5 μg of the protein extract was incubated in an acetate buffer [10 mM sodium acetate, pH 5.0], supplemented with 1 mM dithiothreitol (DTT) in a final volume of 100 μL. The reaction used a fluorogenic peptide substrate, *N*-benzoyloxycarbonyl-l-phenylalanyl-l-arginine 7-amino-4-methylcoumarin (Z-FR-AMC), at a concentration of 60 μM. Incubation was conducted at 37 °C for 90 min, with relative fluorescence changes measured using a SpectraMax M2e spectrophotometer (Molecular Devices, Sunnyvale, CA, USA). Inhibition assays were performed using three concentrations (33.3, 100, and 300 µM) of the promising candidate and trans-epoxysuccinyl-L-leucylamido(4-guanidino)butane (E-64), each analyzed separately. A control was included using a low DMSO concentration (≤1%). Enzymatic activity was expressed in µmol/min/mg of protein, with residual activity presented as a percentage.

### 3.10. Statistical Analysis

Statistical analysis was performed using GraphPad Prism version 8.2.1. The dataset underwent a One-Way ANOVA, followed by the Kruskal–Wallis test for non-parametric comparisons. A *p*-value threshold of 0.05 was established to denote statistical significance.

## 4. Conclusions

The new pyrazole-imidazoline derivatives (**1a−m**) were optimized based on a previously identified hit compound described as a cysteine protease inhibitor [29]. This new series exhibited favorable physicochemical properties and was predicted to have oral bioavailability, along with low cytotoxicity in mammalian cells. Overall, they demonstrated activity against the intracellular forms of *T. cruzi*, with **1k** emerging as the most promising candidate, standing out for its activity (IC_50_ = 3.3 ± 0.2 µM), selectivity (SI = 73.9), and potency (pIC_50_ = 5.4). Structural modifications, including the removal of the amino group (NH_2_) and the introduction of a trifluoromethyl (CF_3_) substituent at the *para* position of the phenyl ring, positively modulated antiparasitic activity. Compound **1k** effectively permeated 3D cardiac microtissues and significantly reduced parasite burden. Although it exhibited an effect similar to Bz in preventing the reactivation of infection, neither compound achieved complete sterilization of cultures after 10 days of treatment. The combination of **1k** and Bz revealed an additive interaction, suggesting a beneficial outcome for in vivo combination therapy. Finally, structural alterations in **1k** resulted in the loss of its ability to inhibit *T. cruzi* cysteine protease, implying that a distinct mechanism of action may be responsible for its antiparasitic efficacy. Together, these data underscore the optimization of pyrazole-imidazoline derivatives and suggest their potential as promising candidates in the development of anti-*T. cruzi* agents.

## Figures and Tables

**Table 1:** Physicochemical properties of the pyrazole-imidazoline derivatives.

Compounds	MW	cLogP	cLogS	HBA	HBD	TPSA	Drug Likeness	RB
**1a**	296.16	1.085	−3.63	5	2	68.23	6.54	2
**1b**	245.26	−0.02	−2.74	5	2	68.23	5.2	2
**1c**	275.74	0.823	−3.23	5	2	68.23	6.46	2
**1d**	275.74	0.823	−3.23	5	2	68.23	6.46	2
**1e**	241.29	0.21	−2.5	5	2	68.23	6.45	2
**1f**	226.28	0.543	−1.9	4	1	42.21	6.24	2
**1g**	241.29	0.217	−2.5	5	2	68.23	6.45	2
**1h**	226.28	0.543	−1.9	4	1	42.21	6.24	2
**1i**	295.26	0.721	−2.93	5	2	68.23	−0.64	3
**1j**	295.26	0.721	−2.93	5	2	68.23	−0.64	3
**1k**	280.25	1.04	−2.33	4	1	42.21	−0.83	3
**1l**	363.26	1.57	−3.71	5	2	68.23	−0.64	4
**1m**	348.24	1.89	−3.11	4	1	42.21	−0.83	4
**Bz**	260.25	−0.25	−1.62	7	1	92.74	−1.71	5

The physicochemical parameter values of pyrazole-imidazoline derivatives were calculated using the SMILES representation of each molecule using Osiris DataWarrior software (version 5.5.0). MW: molecular weight, cLogP: octanol–water partition coefficient (lipophilicity), cLogS: water solubility, HBA: number of hydrogen bond acceptors, HBD: number of hydrogen bond donors, TPSA: topological polar surface area, RB: rotatable bond.

**Table 2:** Cytotoxicity and anti-*Trypanosoma cruzi* activity of pyrazole-imidazoline derivatives.

Compounds(R)	Anti-*T. cruzi* Activity (Mean ± SD)						Cytotoxicity (Mean ± SD)
	Trypomastigotes		Intracellular Amastigotes				
	IC_50_ (µM)	IC_90_ (µM)	IC_50_ (µM)	IC_90_ (µM)	SI	pIC_50_	CC_50_ (µM)
**1a** (2,4-diCl)	>100	>100	40.8 ± 3.0	>100	>12.2	4.12	>500
**1b** (3-F)	>100	>100	>100	>100	ND	<4	>500
**1c** (4-CH_3_)	>100	>100	>100	>100	ND	<4	>500
**1d** (4-CH_3_) *	>100	>100	33.9 ± 0.8	>100	>14.7	4.4	>500
**1e** (3-Cl, 4-CH_3_)	76.5 ± 10.3	>100	68.9 ± 8.1	96.6 ± 0.2	>7.2	4.1	>500
**1f** (4-Cl, 2-CH_3_)	>100	>100	76.1 ± 1.2	>100	>6.5	4.1	>500
**1g** (3-CH_3_)	>100	>100	78.9 ± 2.2	>100	>6.3	4.1	>500
**1h** (3-CH_3_) *	>100	>100	26.2 ± 3.4	89.7 ± 7.4	>19.1	4.6	>500
**1i** (3-CF_3_)	>100	>100	51.9 ± 3.6	>100	>9.6	4.2	>500
**1j** (4-CF_3_)	>100	>100	88.1 ± 0.2	>100	>5.6	4.0	>500
**1k** (4-CF_3_) *	72.2 ± 10.3	>100	3.3 ± 0.2	28.1 ± 0.4	73.9	5.4	243.8 ± 21.1
**1l** (3,5-diCF_3_)	>100	>100	27.7 ± 2.0	80.1 ± 11.4	16.7	4.5	463.6 ± 15.2
**1m** (3,5-diCF_3_) *	>100	>100	32.4 ± 5.9	80.7 ± 0.4	3.5	4.5	113.5 ± 11.9
**Bz**	20.5 ± 1.8	>100	2.2 ± 0.4	10.4 ± 0.5	>221.2	5.6	>500

The mean values for IC_50_, IC_90_, and CC_50_ were obtained from three independent experiments and are presented as the average ± standard deviation (SD); IC_50_ refers to the concentration that inhibits 50% of the parasite’s viability, measured at 24 h for trypomastigotes and 72 h for intracellular amastigotes; CC_50_ indicates the concentration that reduces Vero cell viability by 50%; the selectivity index (SI) = CC_50_/IC_50_; not determined (ND). The asterisk (*) represents derivatives that do not contain an amino group (NH_2_).

## Data Availability

The authors confirm that the data supporting the findings of this study are available within the article and its Supplementary Materials.

## Supplementary Materials

The following supporting information can be downloaded at: https://www.mdpi.com/article/10.3390/molecules30153082/s1, NMR spectra of the compounds.
