# Supplementary material for: Anti-Trypanosoma cruzi Potential of New Pyrazole-Imidazoline Derivatives
Source: Molecules. 2025 Jul 23;30(15):3082. doi: 10.3390/molecules30153082 (PMC12348844; doi:10.3390/molecules30153082)

^{1}H NMR of **1a**

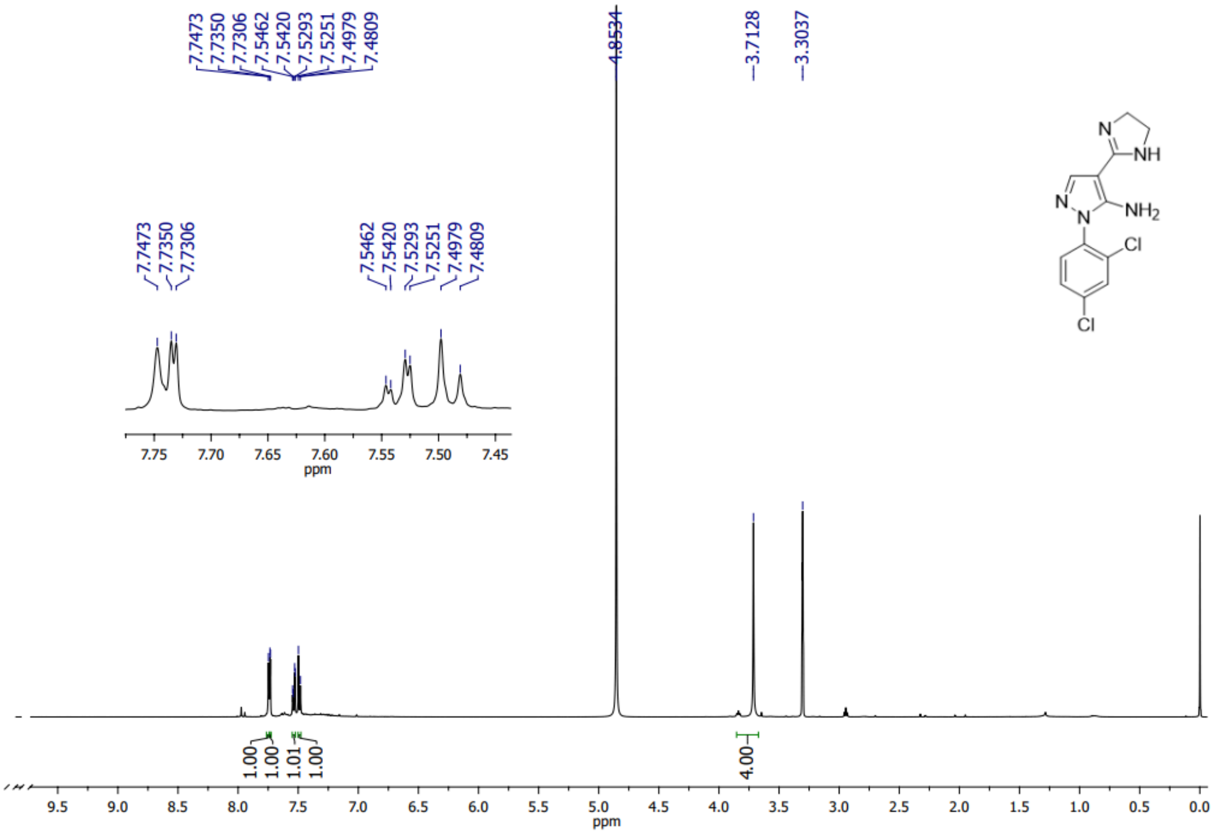

^{13}C NMR of **1a**

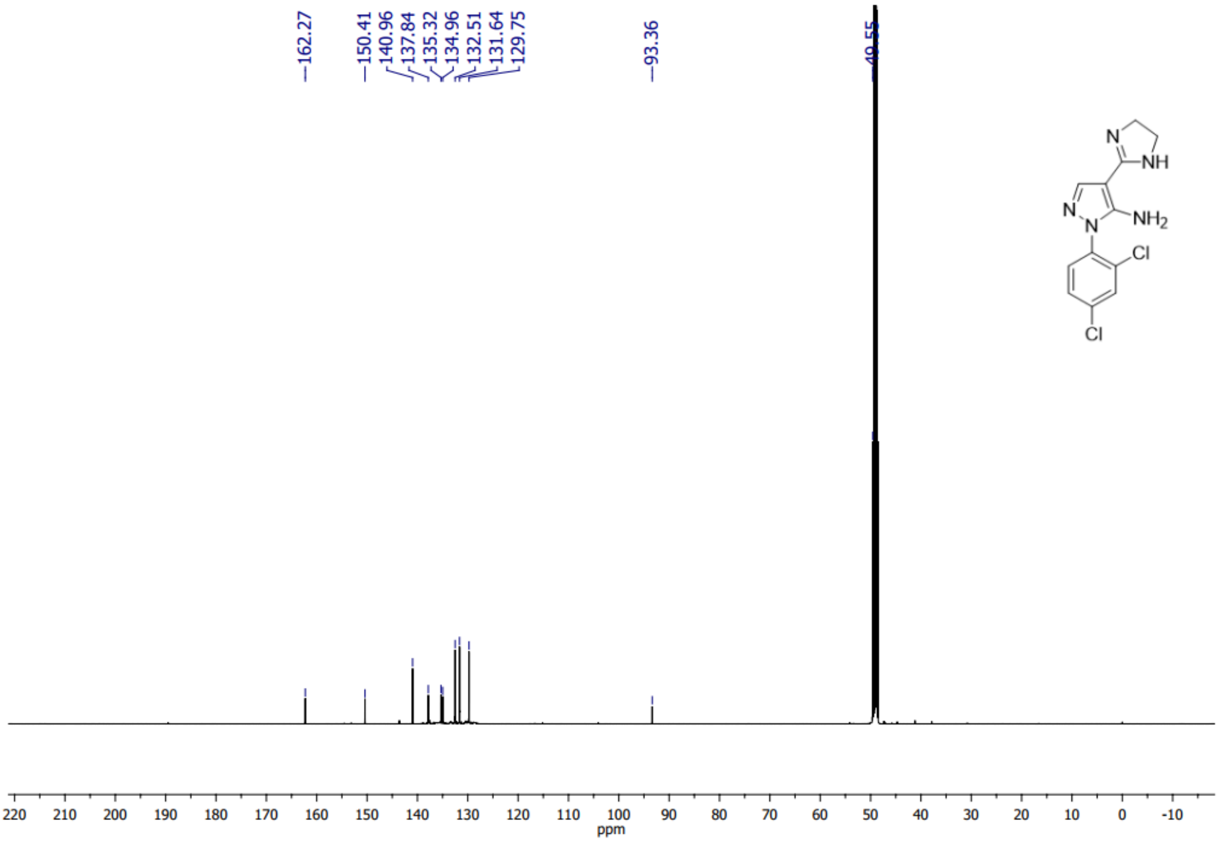

HSQC spectrum of **1a**

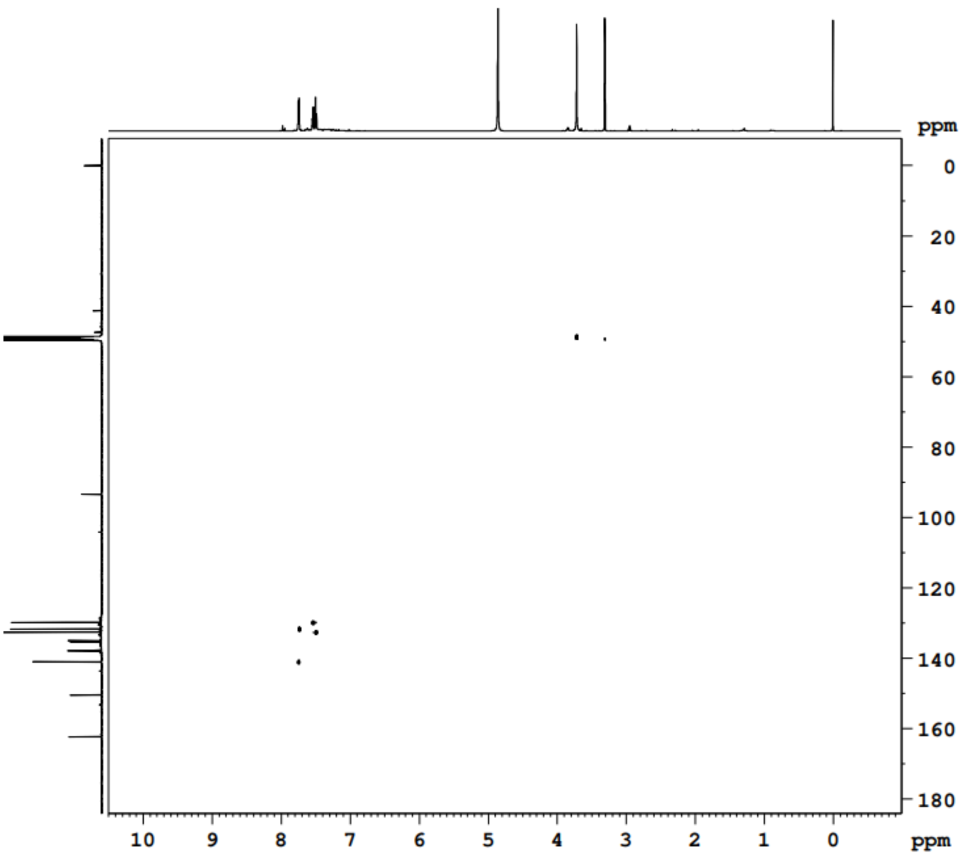

HSQC expansion of **1a**

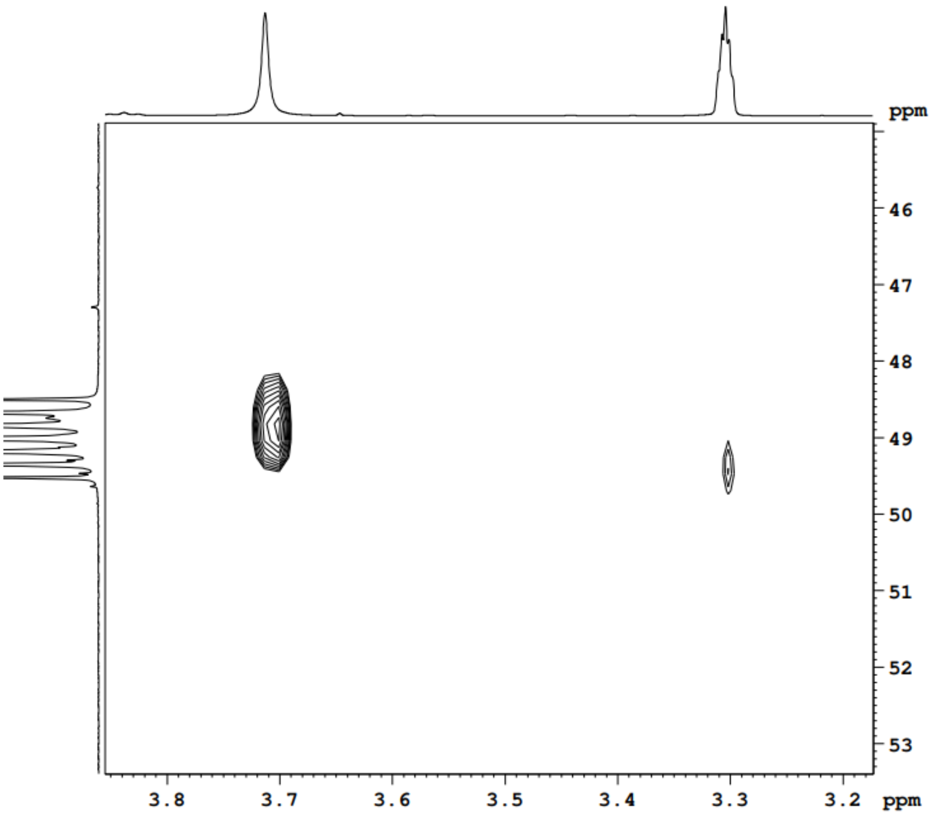

^{1}H NMR of **1b**

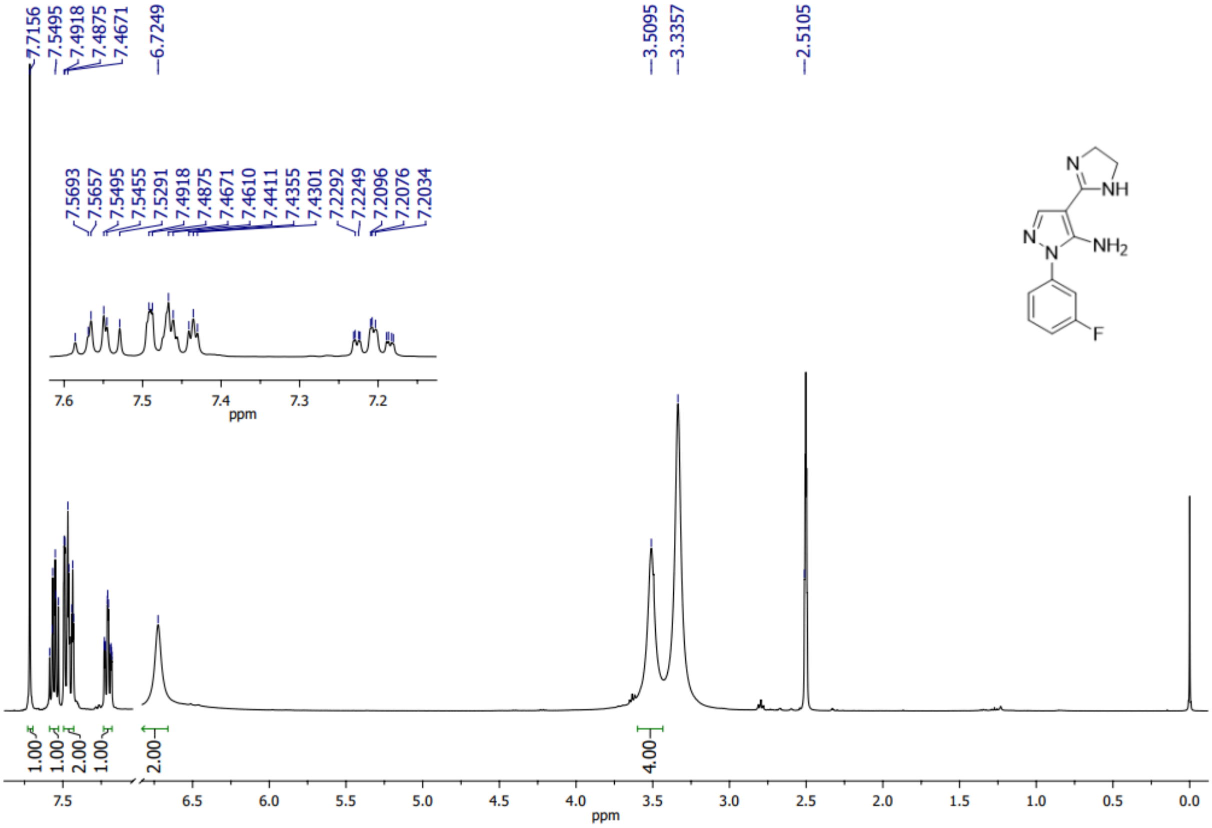

^{13}C NMR of **1b**

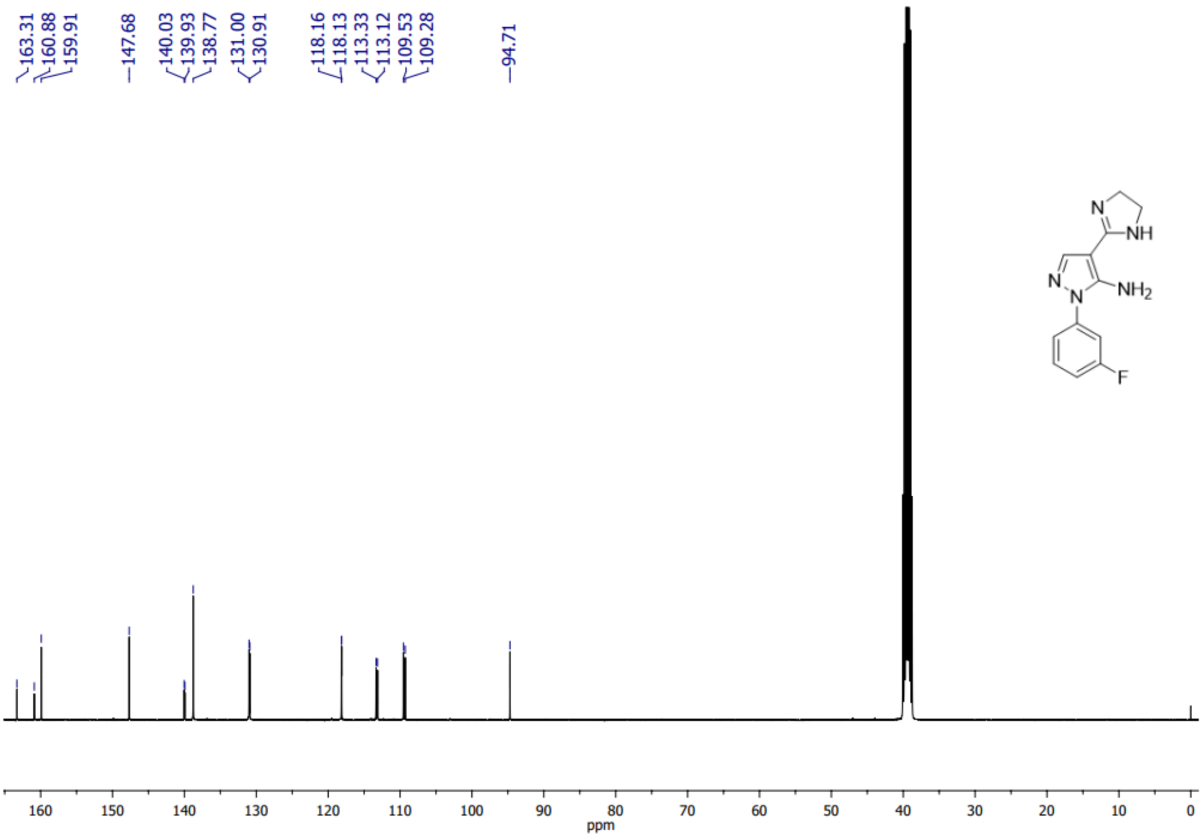

^{1}H NMR of **1c**

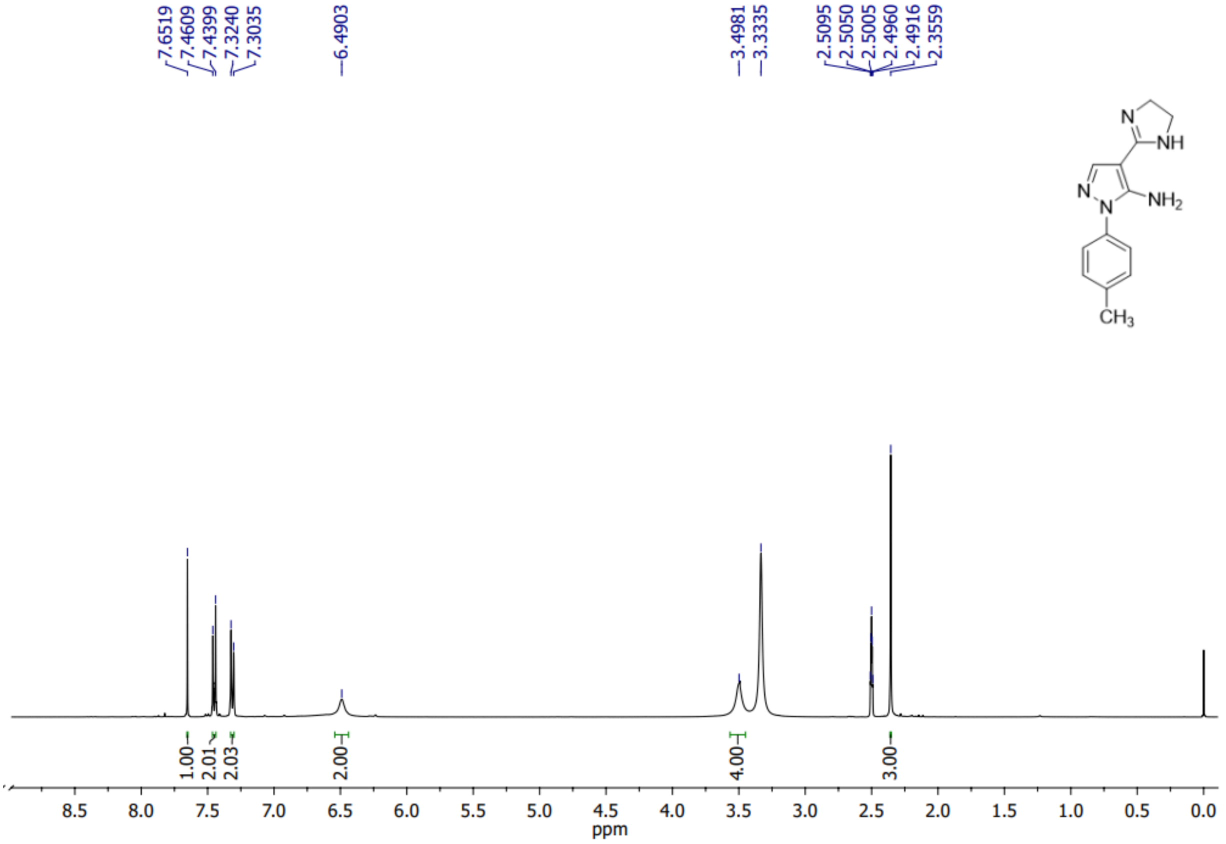

^{13}C NMR of **1c**

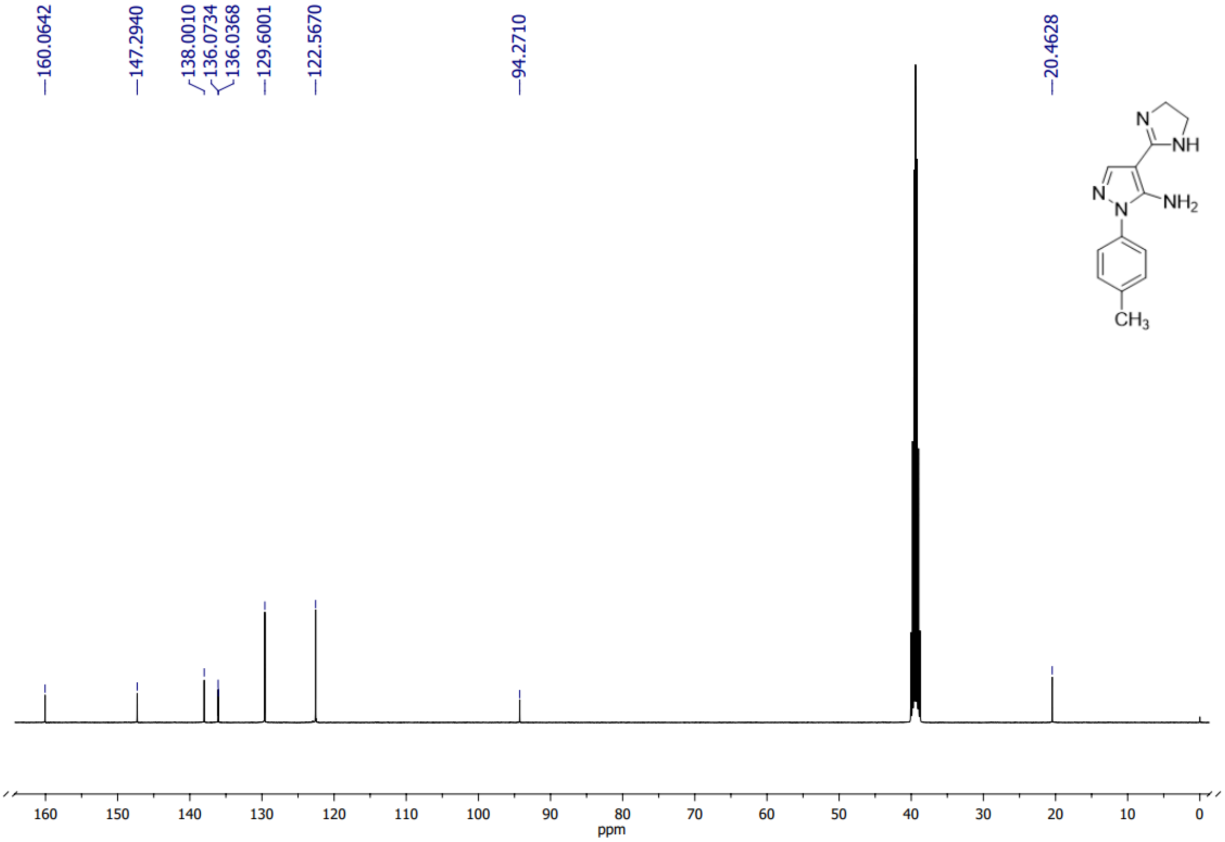

^{1}H NMR of **1d**

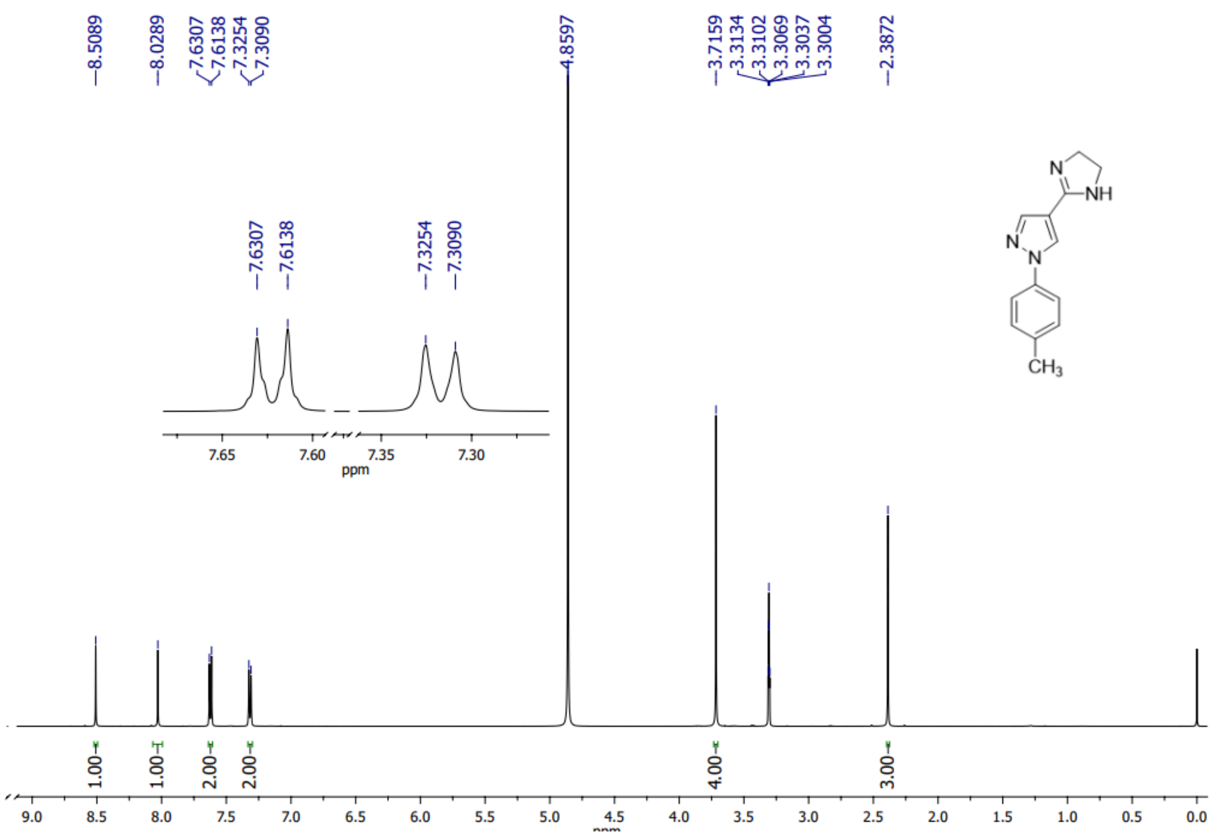

^{13}C NMR of **1d**

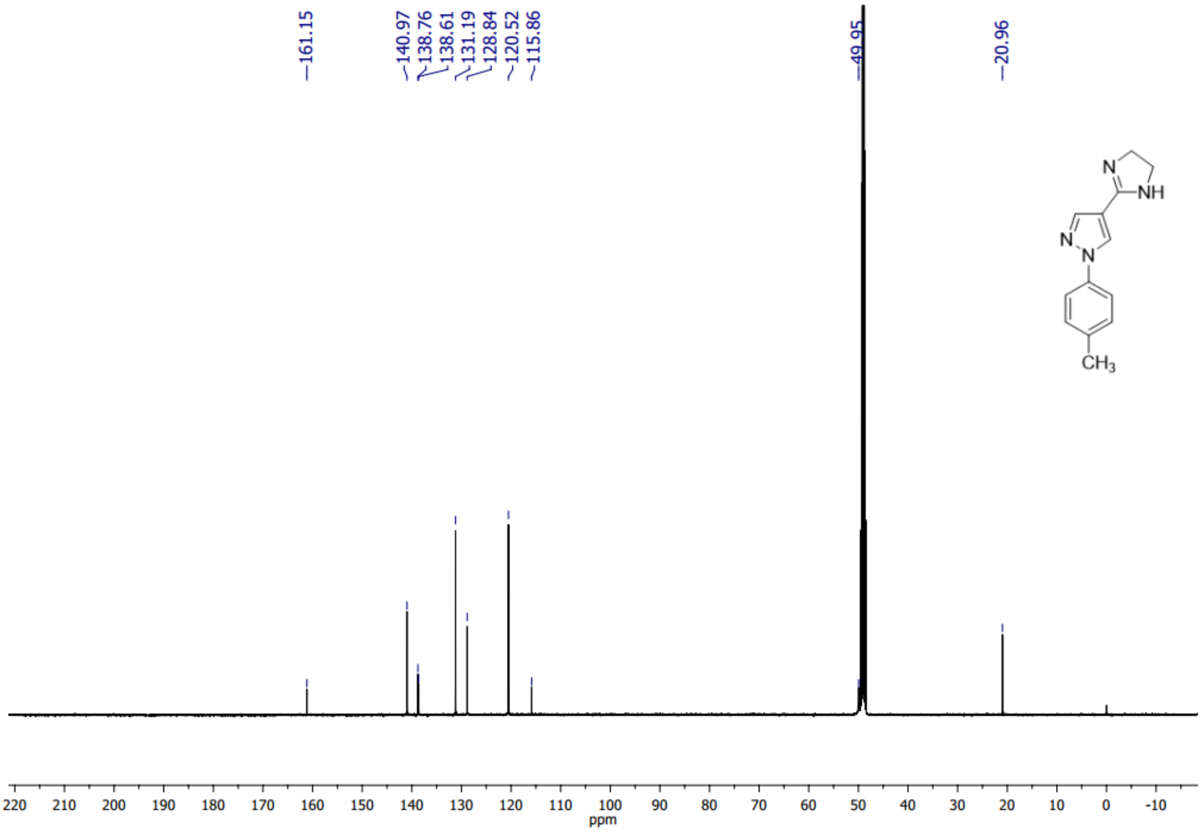

^{1}H NMR of **1e**

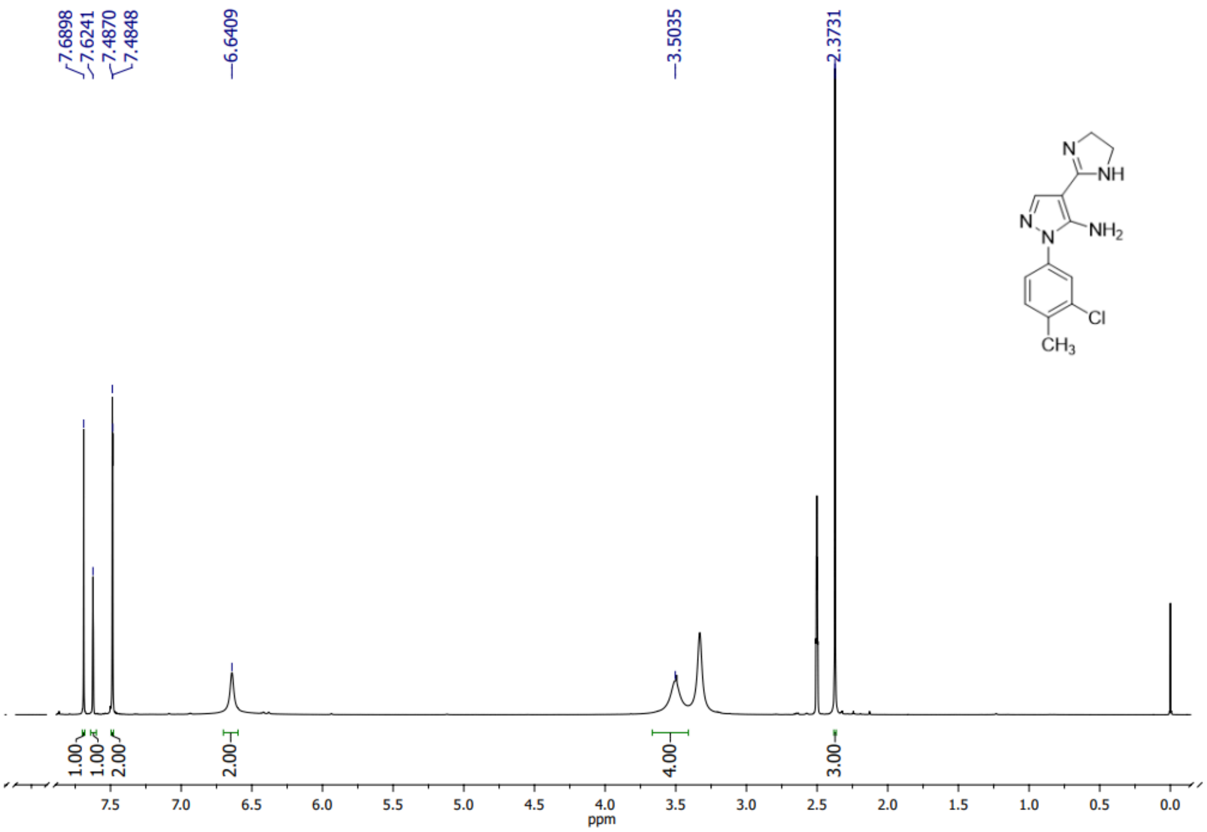

^{13}C NMR of **1e**

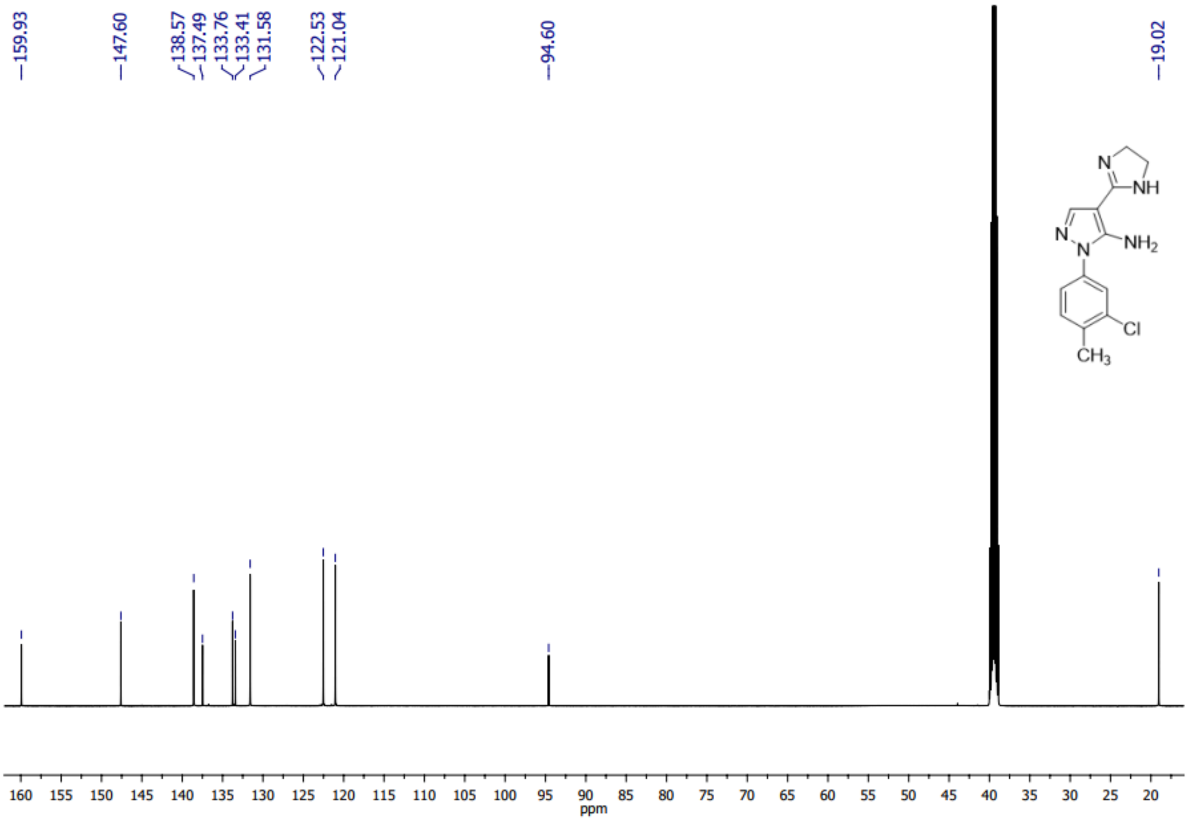

^{1}H NMR of **1f**

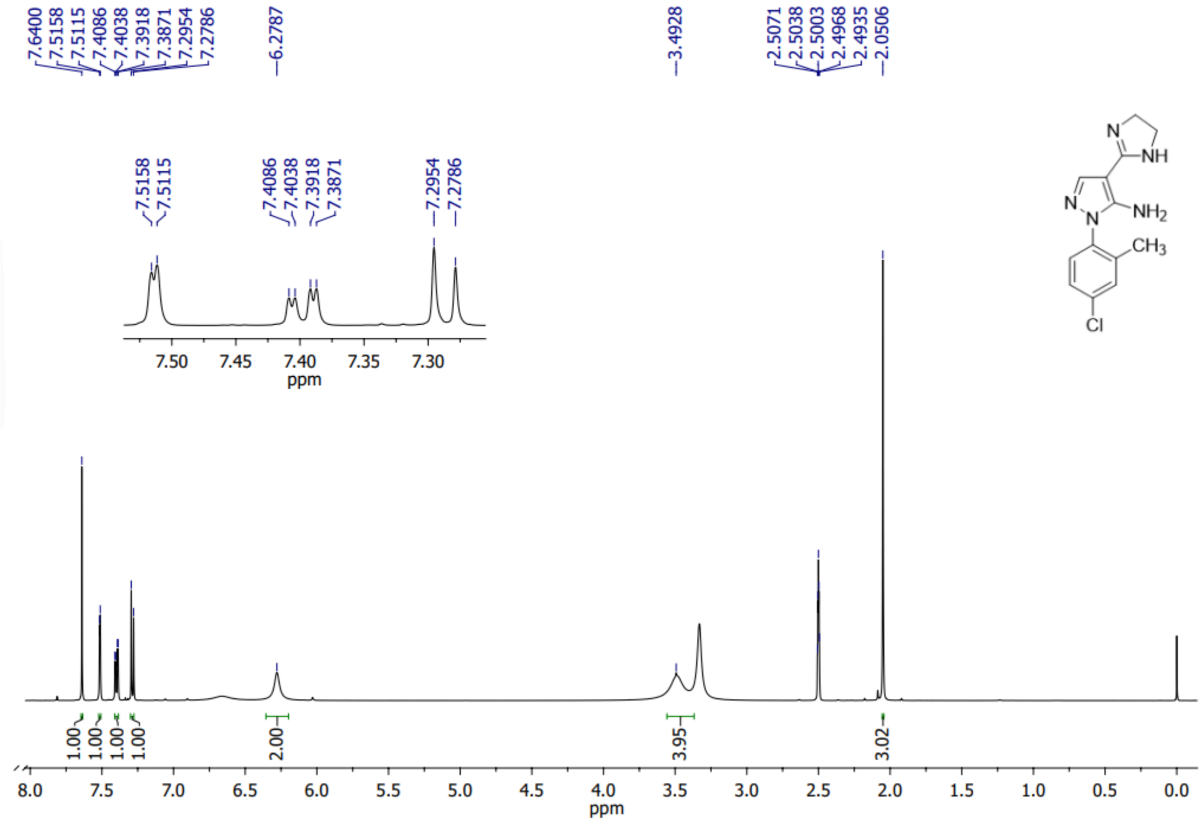

^{13}C NMR of **1f**

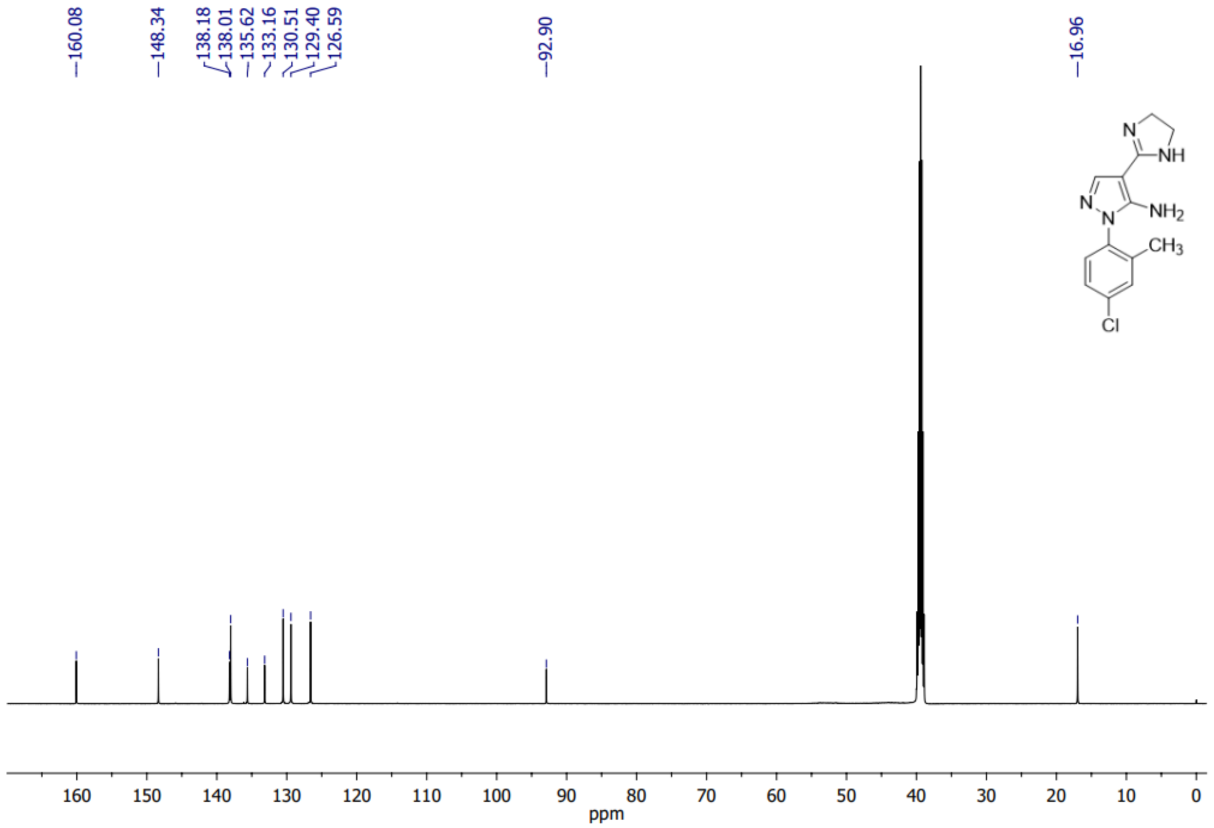

^{1}H NMR of **1g**

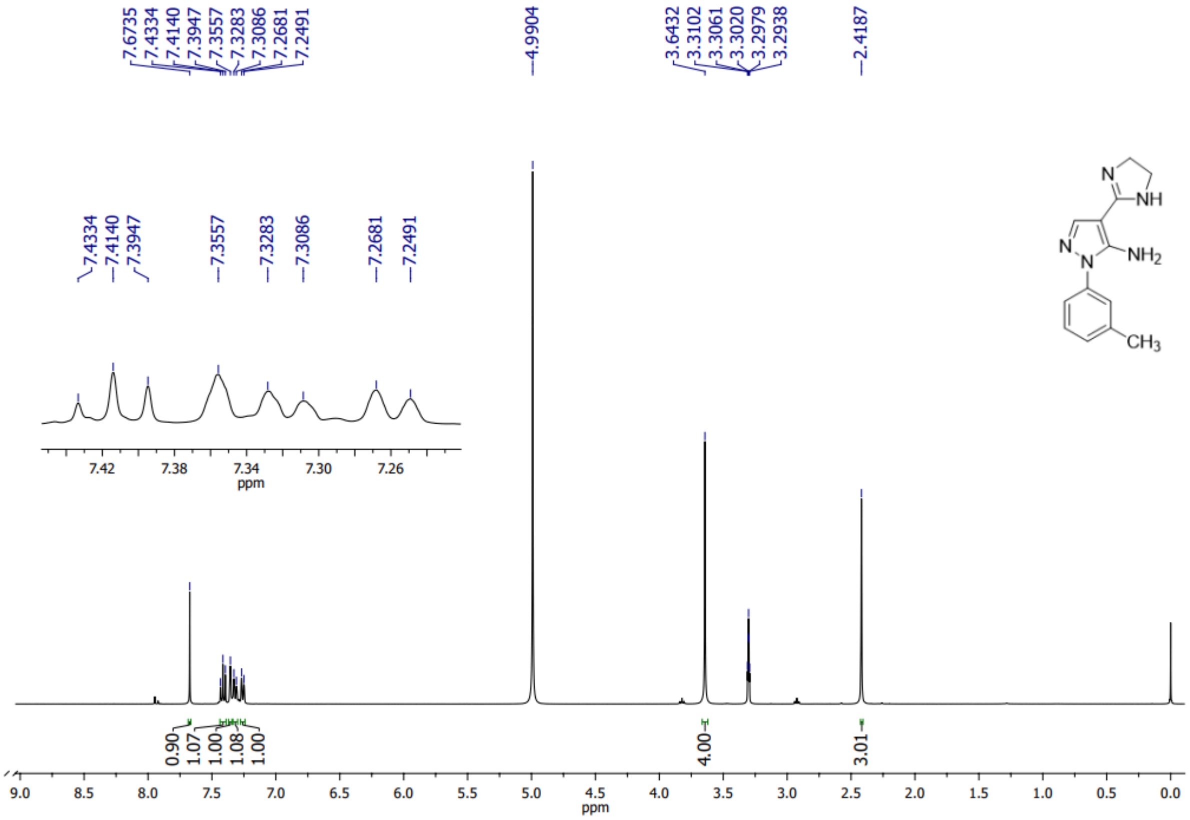

^{13}C NMR of **1g**

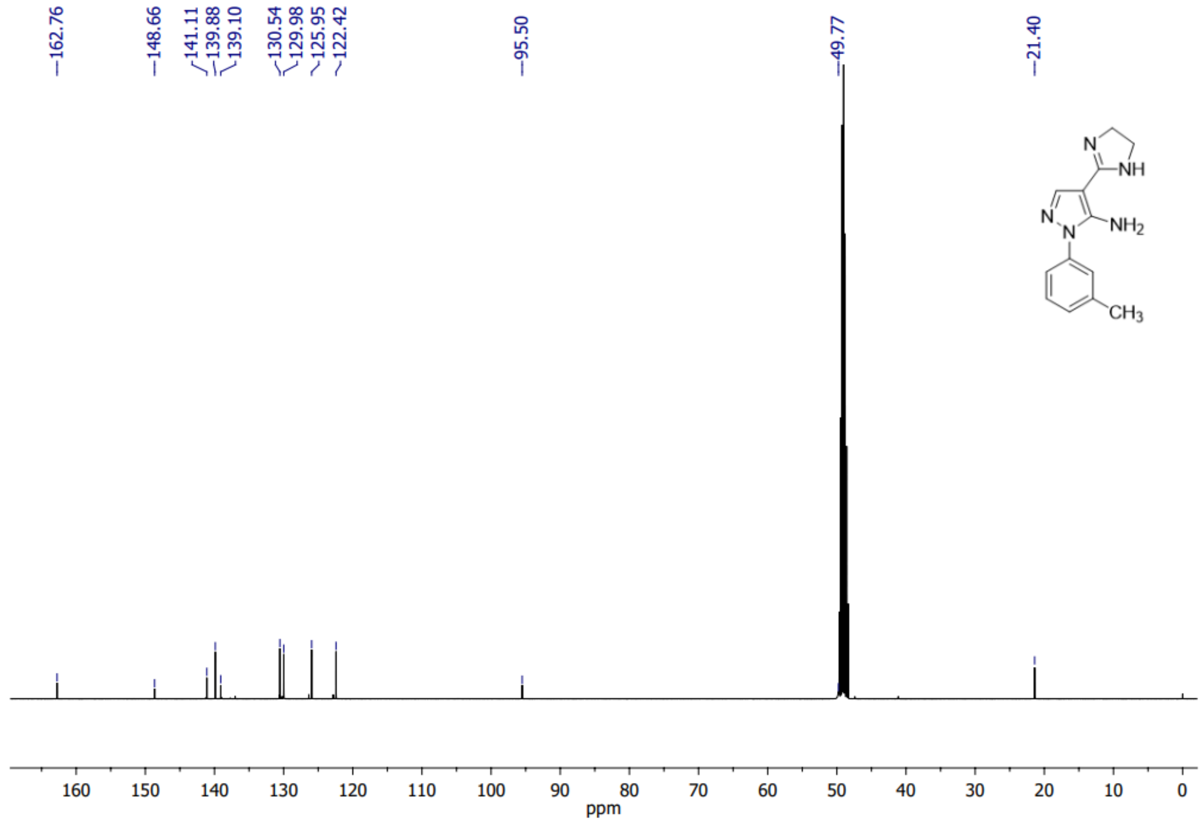

^{1}H NMR of **1h**

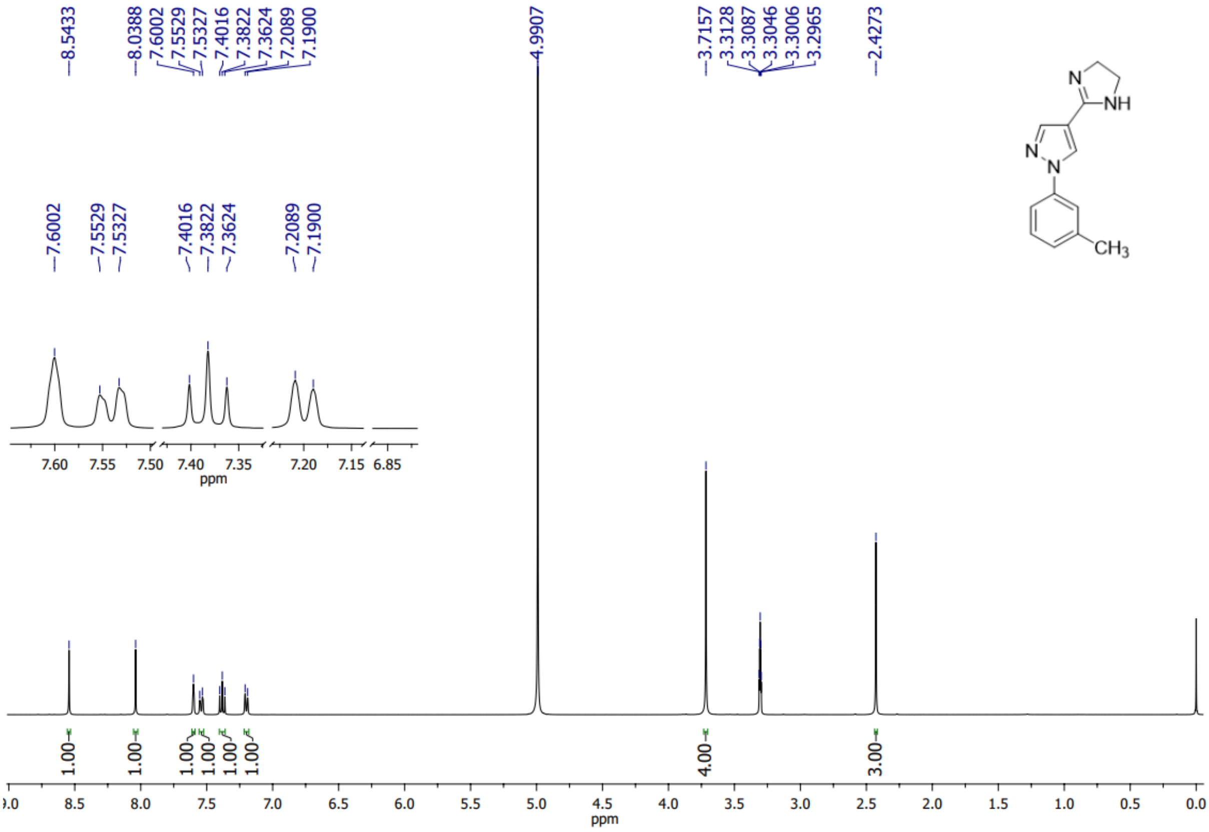

^{13}C NMR of **1h**

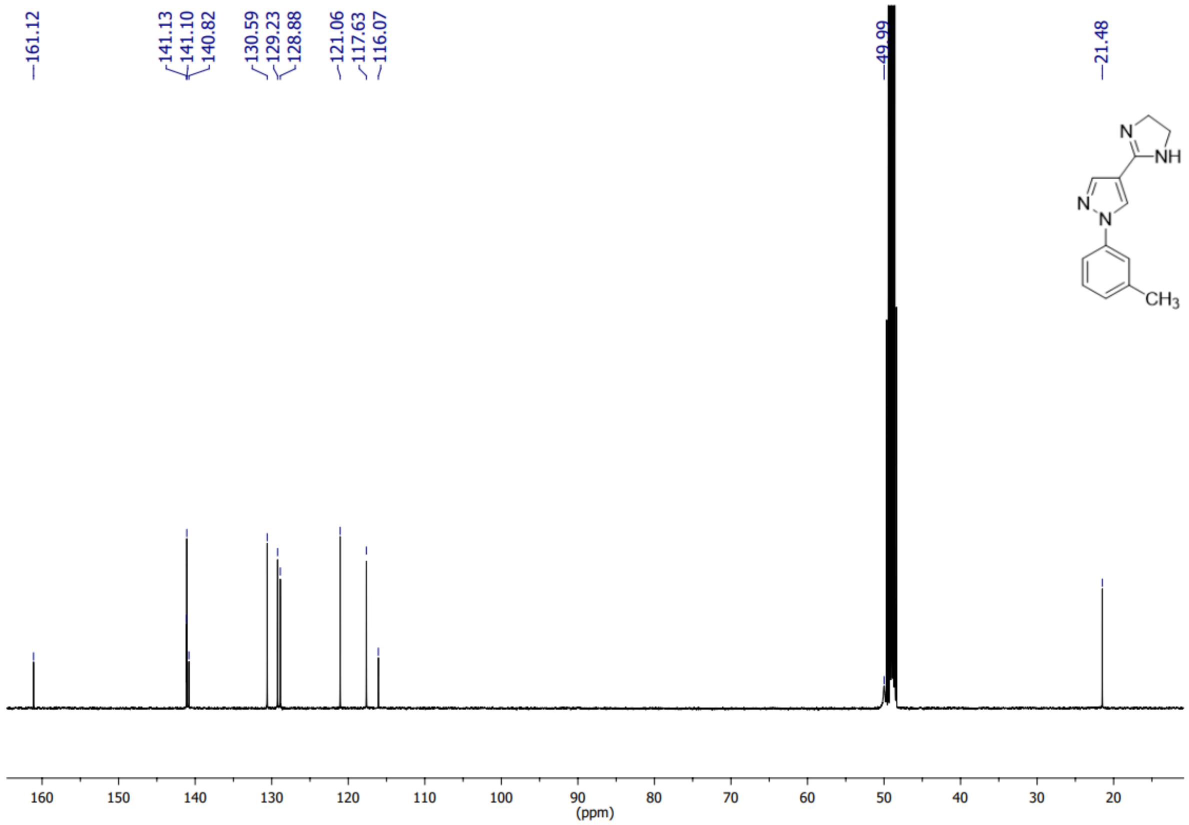

^{1}H NMR of **1i**

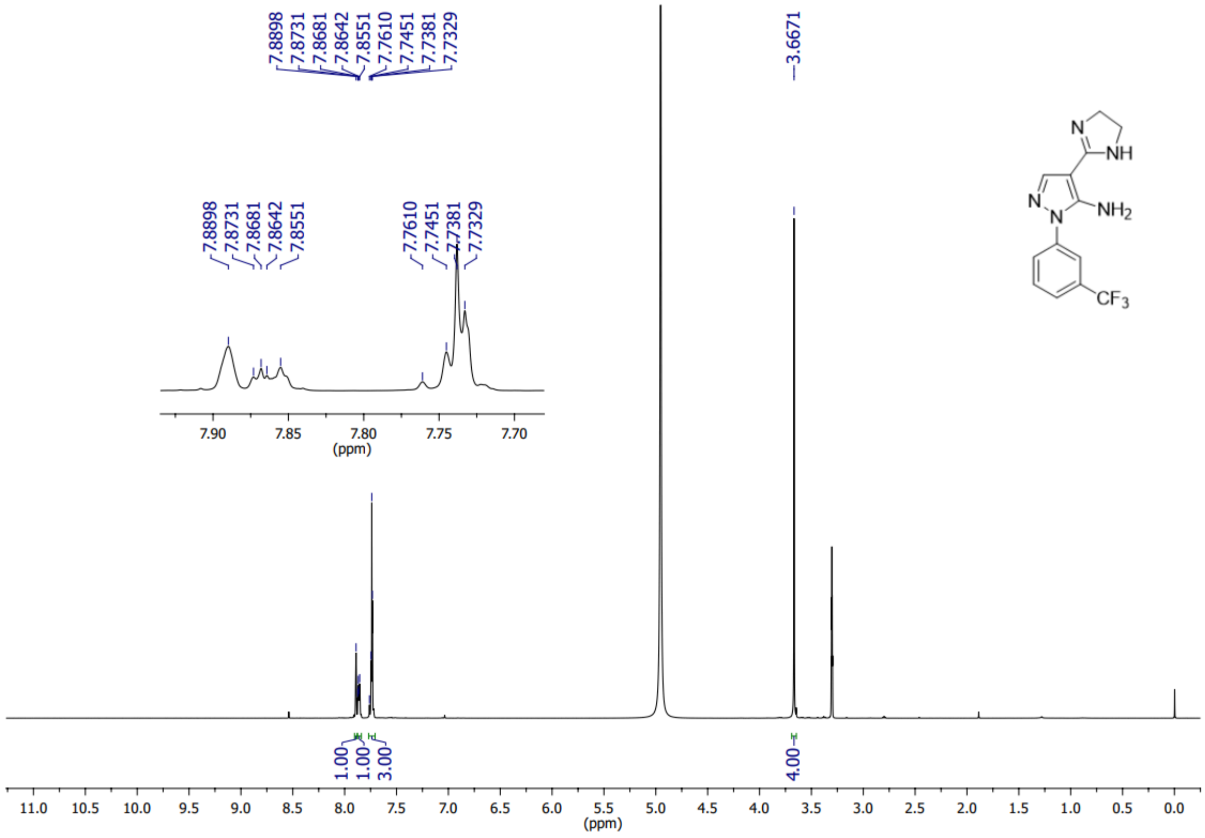

^{13}C NMR of **1i**

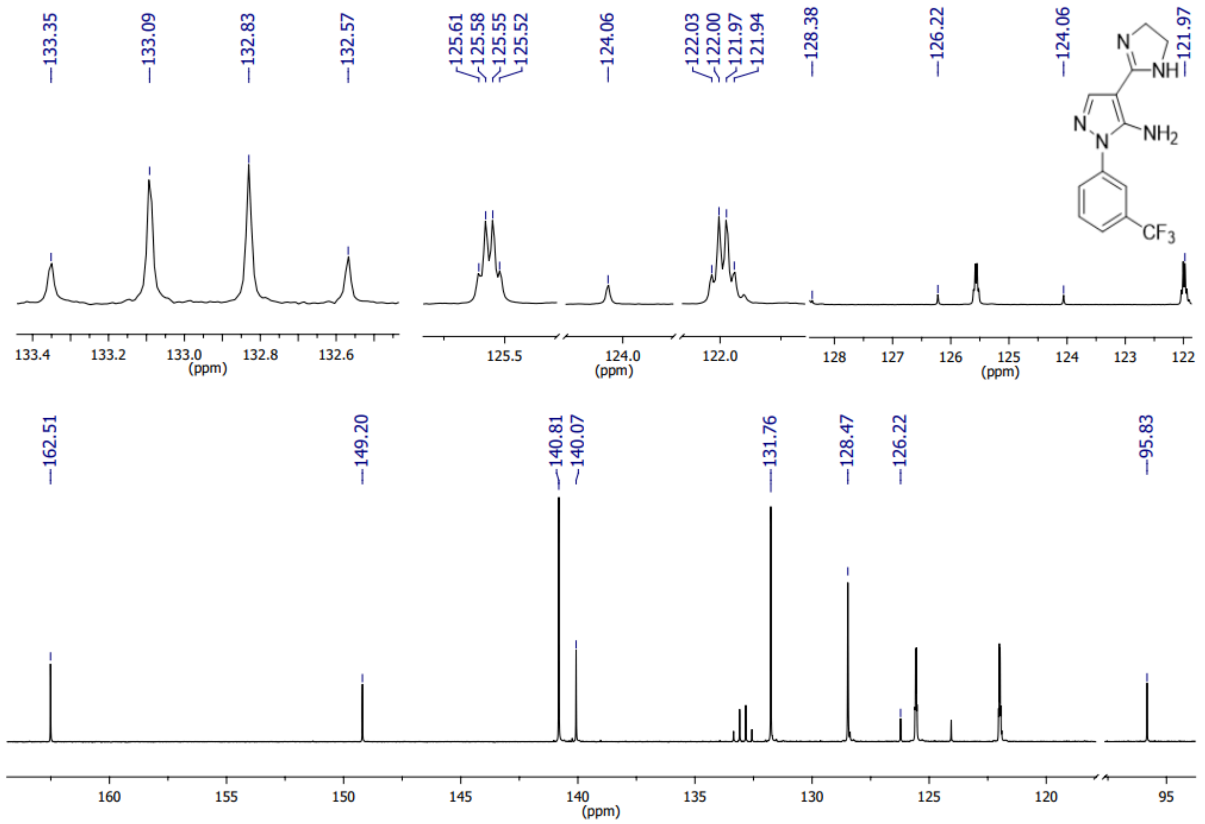

^{1}H NMR of **1j**

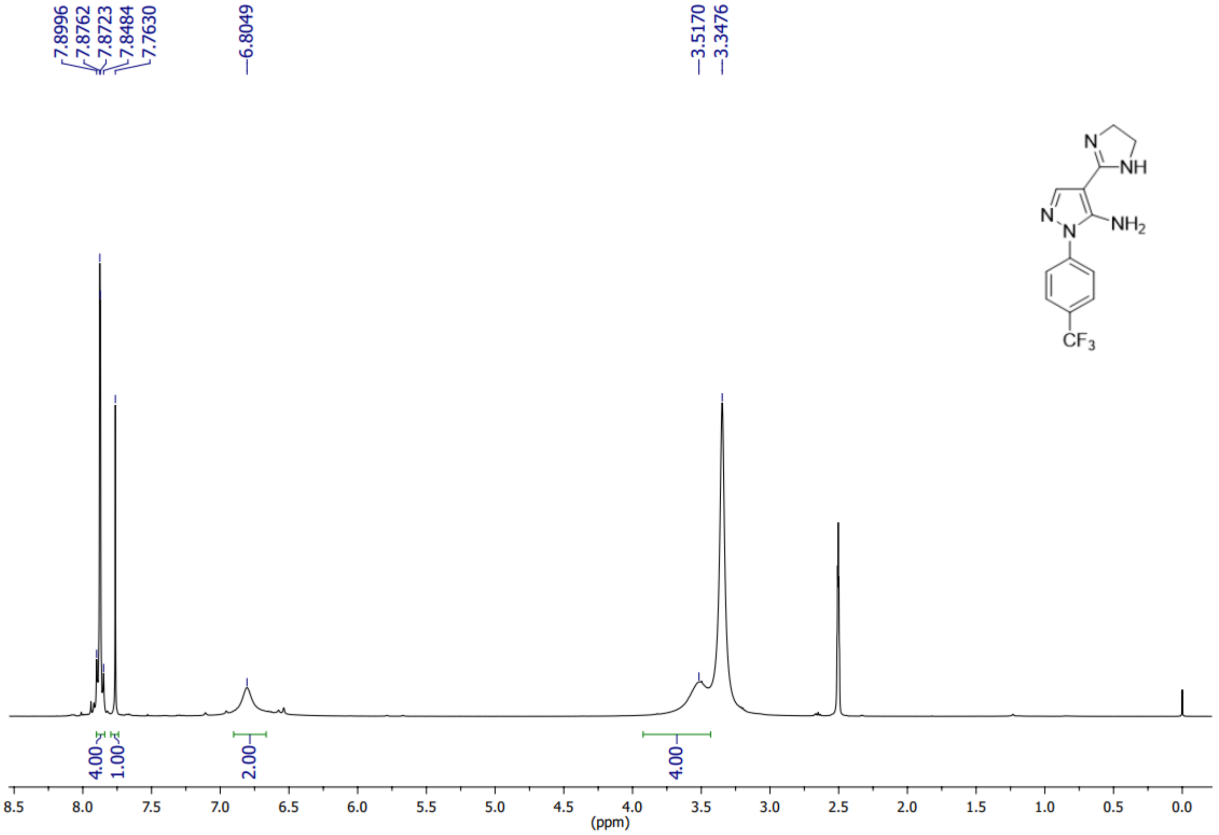

^{13}C NMR of **1j**

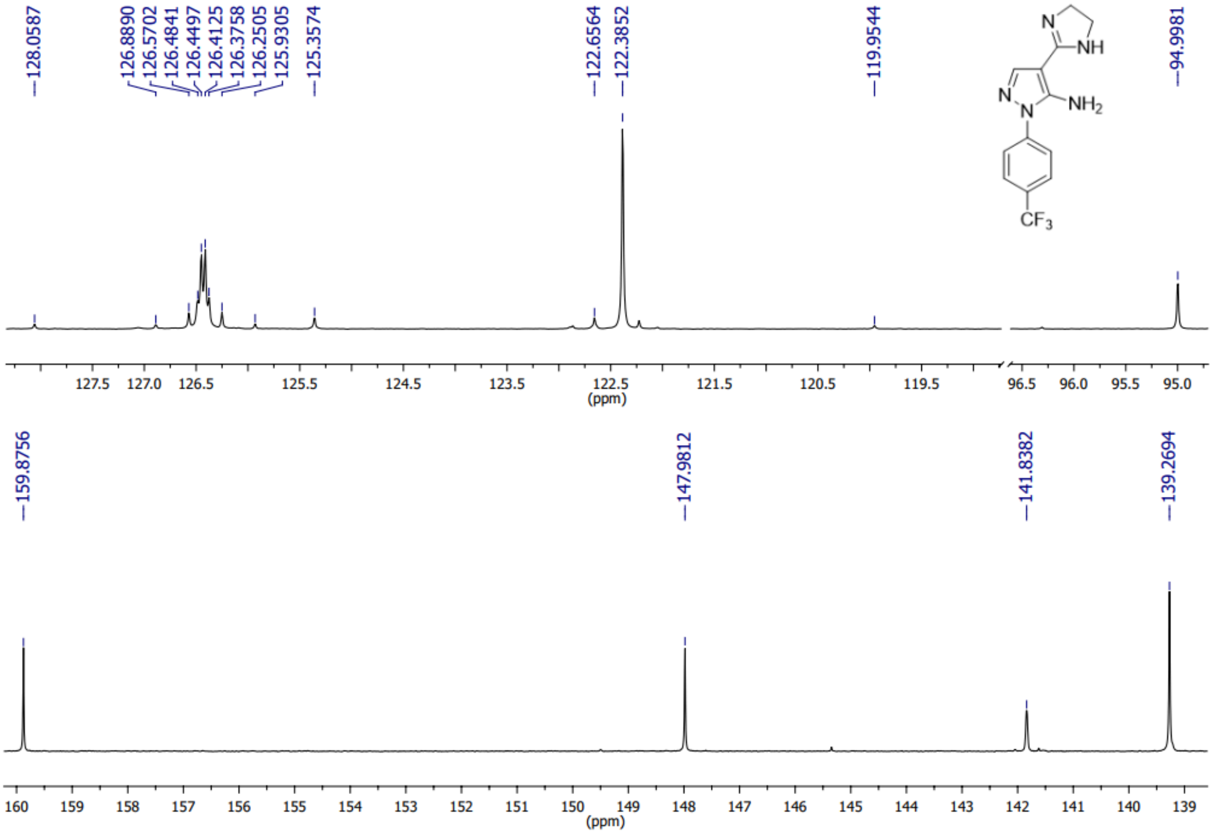

^{1}H NMR of **1k**

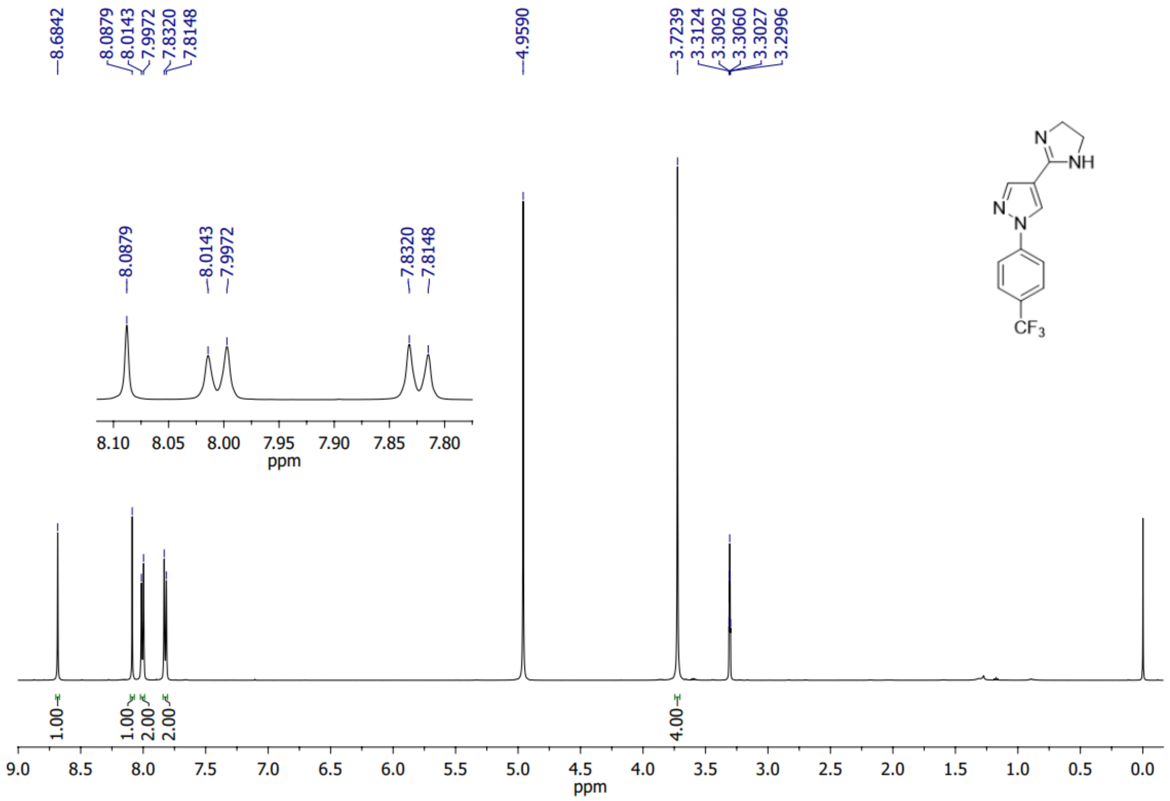

^{13}C NMR of **1k**

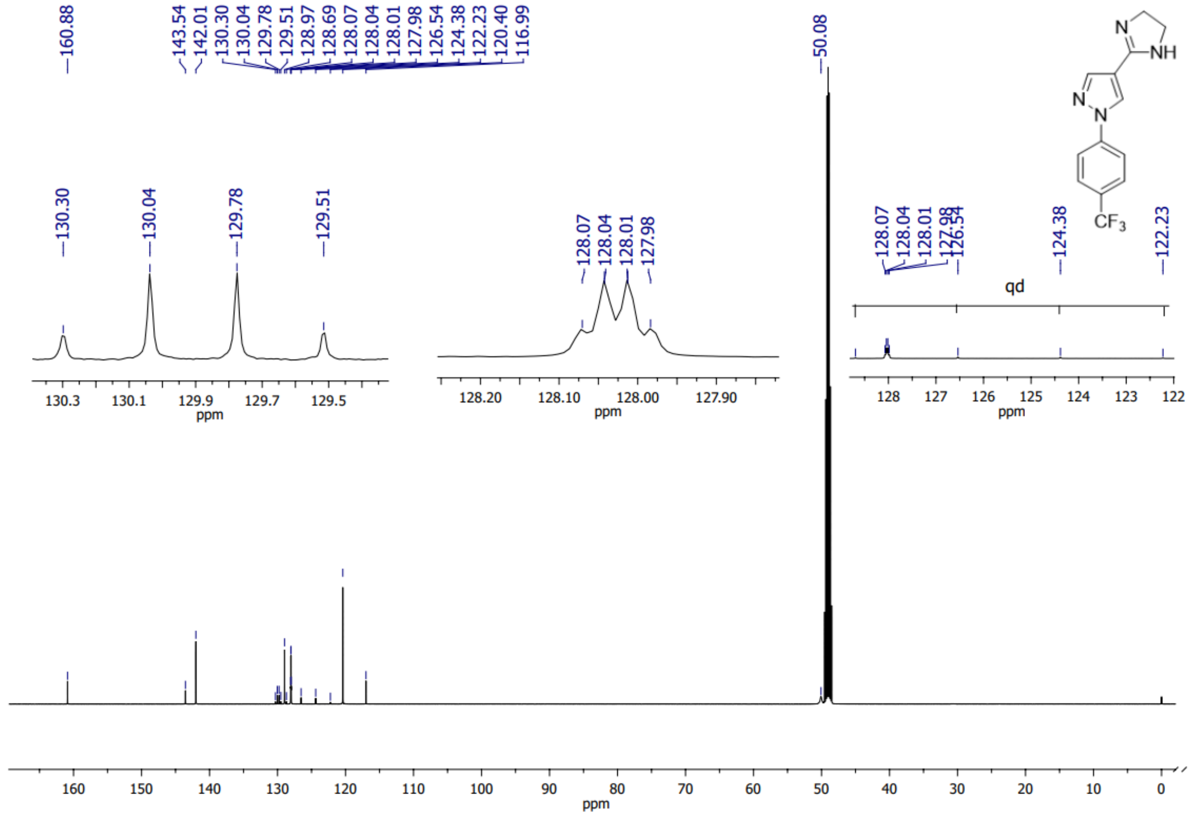

^{1}H NMR of **1l**

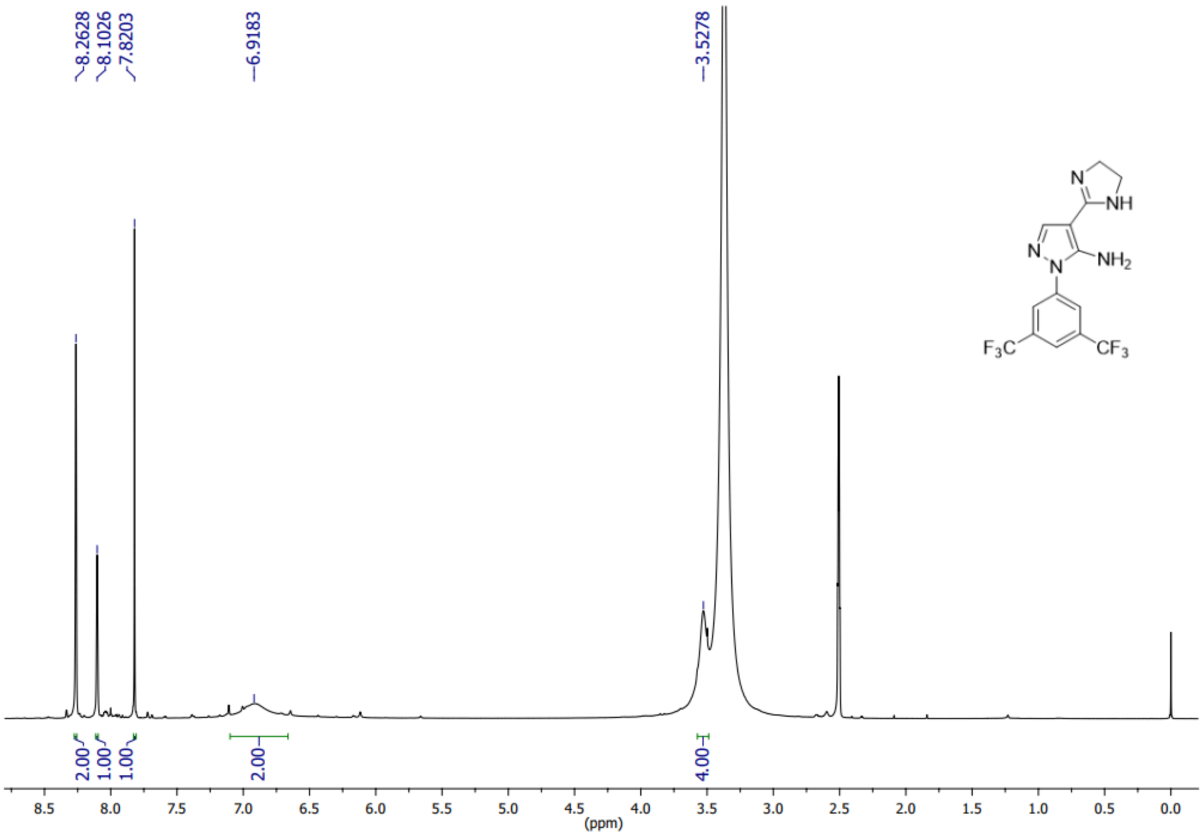

^{13}C NMR of **1l**

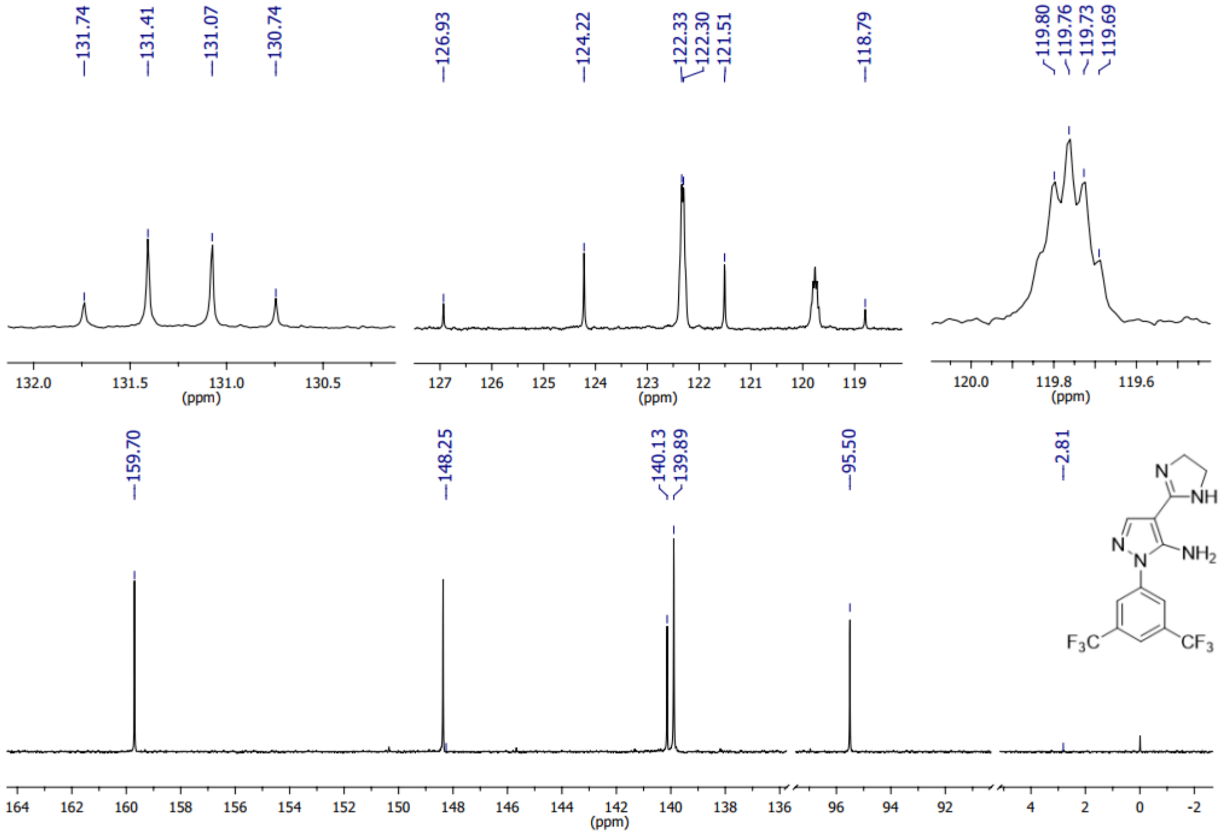

^{1}H NMR of **1m**

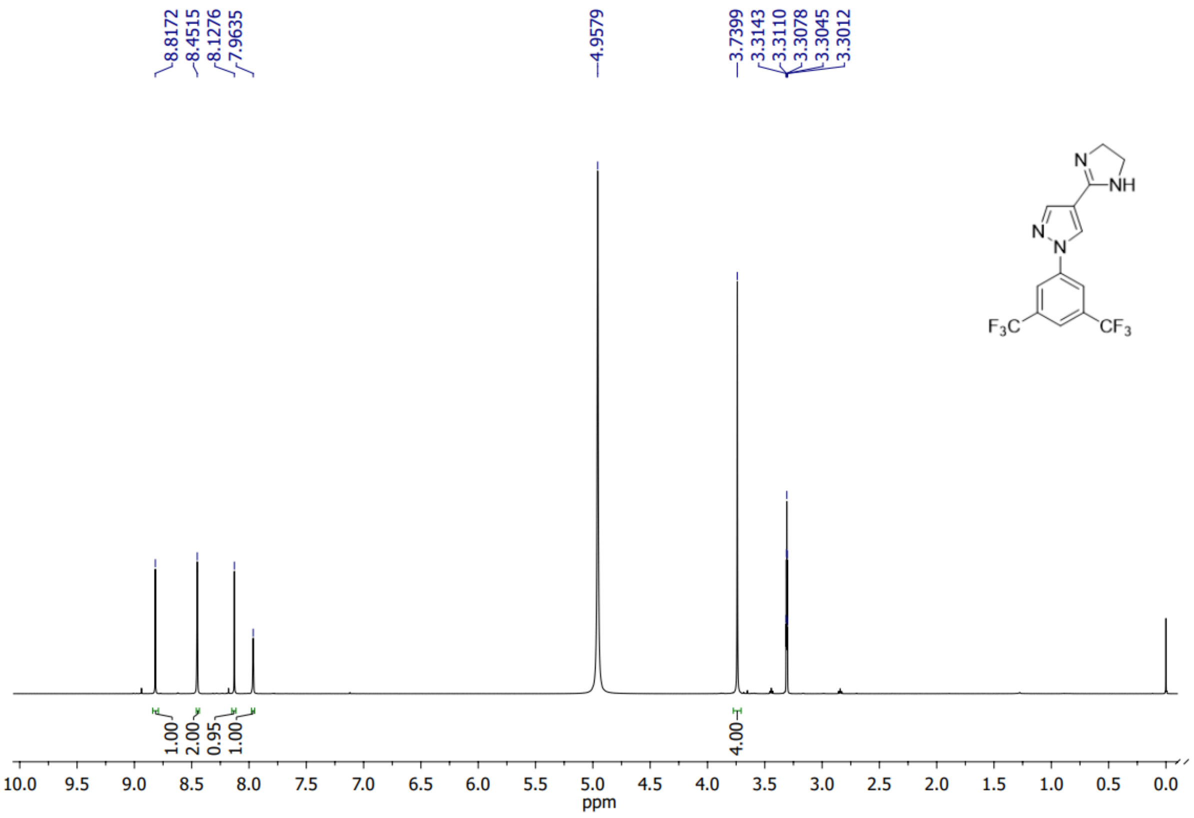

^{13}C NMR of **1m**

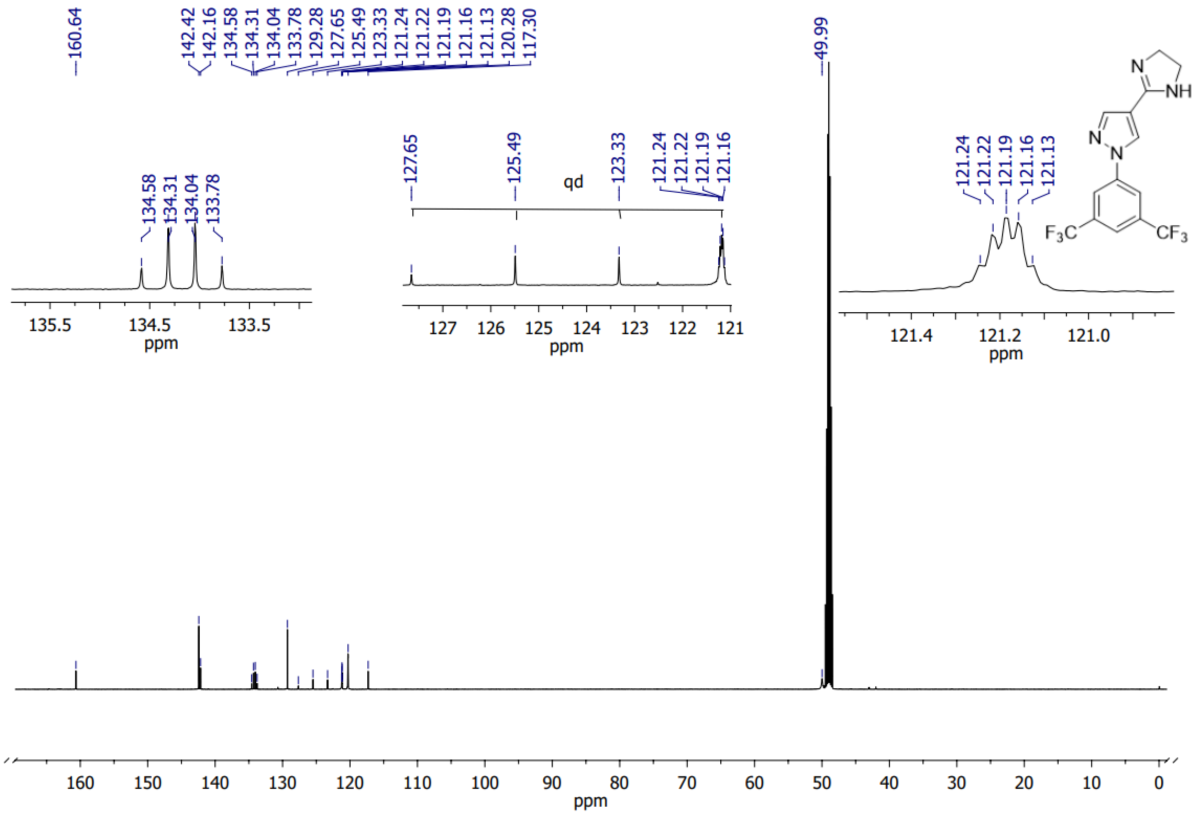

Supplement: Supplementary file 1 [file molecules-30-03082-s001.zip › molecules-3750989-supplementary.pdf]
